# Supplementary material for: Health-related quality of life, functional impairment and comorbidity in people with mild-to-moderate chronic kidney disease: a cross-sectional study
Source: BMJ Open. 2020 Aug 6;10(8):e040286. doi: 10.1136/bmjopen-2020-040286 (PMC7412591; doi:10.1136/bmjopen-2020-040286)
Supplement: Supplementary data [file bmjopen-2020-040286supp001.pdf]

Supplementary material

Table S1. Characteristics of those with and without complete follow up data at five years.

	**Complete HRQoL data (n=1008)**	**Incomplete HRQoL data (n=486)**
**Age (mean(SD))**	70.6 (8.6)	74.7 (9.0)
**Sex (n(%) male)**	387 (38)	160 (33)
**Index of multiple deprivation (IMD) (n (%) in most deprived quintile)**	82 (8)	49 (10)
**Comorbidities (n (%) with three or more)**	344 (34)	206 (42)
**Functional impairment (KPS ≤70)**	234 (23)	2 (0.4%)

Table S2. Associations between patient-reported EQ-5D-5L quality of life domains and clinician-assessed functional status.

**Patient-reported EQ-5D-5L quality of life domain**		**No clinician-assessed functional impairment (KPS score >70)**		**Clinician-assessed functional impairment (KPS score ≤70)**		**Total RRID cohort**		
		**n**	**%***	**n**	**%***	**n**	**%***	**p****
**Mobility**	1 (no problems in walking about)	416	53.8	10	4.3	426	42.3	<0.001
	2 – 5 (some problems)	358	46.2	224	95.7	582	57.7	
**Self care**	1 (no problems washing or dressing)	726	93.8	116	49.6	842	83.5	<0.001
	2 – 5 (some problems)	48	6.2	118	50.4	166	16.5	
**Usual activities**	1 (no problems doing usual activities)	508	65.6	34	14.5	542	53.8	<0.001
	2 – 5 (some problems)	266	34.4	200	85.5	466	46.2	
**Pain/ discomfort**	1 (no pain or discomfort)	270	34.9	26	11.1	296	29.4	<0.001
	2 – 5 (some pain or discomfort)	504	65.1	208	88.9	712	70.6	
**Anxiety/ depression**	1 (not anxious or depressed)	576	74.4	113	48.3	689	68.4	<0.001
	2 – 5 (some anxiety or depression)	198	25.6	121	51.7	319	31.6	
**EQ-5D-3L index score (converted from EQ-5D-5L)**	> median for age and sex	351	45.4	27	11.5	378	37.5	<0.001
	≤ median for age and sex	423	54.7	207	88.5	630	62.5	
**Total**		774	100.0	234	100.0	1008	100.0	

Fraser SDS, *et al. BMJ Open* 2020; 10:e040286. doi: 10.1136/bmjopen-2020-040286

* Column percentages are shown.** Chi-square test was performed. Abbreviations: KPS= Karnofsky Performance Status, RRID= Renal Risk in Derby

Tables S3 to S6. Logistic regression models examining associations between lower quality of life (EQ-5D-5L domains of usual activities, self-care, pain/discomfort and anxiety/depression categorised as ‘no problems’ vs. ‘any problems’) and patient characteristics. N=1008 in univariable models unless otherwise stated, N=1005 for final multivariable logistic regression models

S3. USUAL ACTIVITIES

		Univariable		Multivariable*	
		**OR (95% CI)**	**p**	**OR (95% CI)**	**p**
**Age (years)**		1.03 (1.02 – 1.05)	<0.001	1.02 (1.00 – 1.04)	0.056
**Female sex (vs. male)**		1.05 (0.81 – 1.36)	0.71	-	-
**Index of multiple deprivation (IMD quintile relative to England) (vs. quintile 5: least deprived) [N=1006]**	Quintile 1 (most deprived)	1.45 (0.87 – 2.40)	0.009#	0.83 (0.46 – 1.52)	0.076#
	Quintile 2	1.93 (1.34 – 2.78)		1.64 (1.08 – 2.50)	
	Quintile 3	1.43 (0.97 – 2.12)		1.37 (0.87 – 2.15)	
	Quintile 4	1.25 (0.87 – 1.78)		1.12 (0.74 – 1.69)	
**Number of comorbidities (vs. no comorbidities)**	One	1. 50 (0.77 – 2.91)	<0.001#	1.41 (0.68 – 2.90)	<0.001#
	Two	2.56 (1.32 – 4.97)		1.82 (0.88 – 3.78)	
	Three or more	6.34 (3.28 – 12.25)		4.20 (2.02 – 8.74)	
**Functional status (KPS score) (vs. KPS >70)**	Functional impairment (KPS ≤70)	11.23 (7.59 – 16.64)	<0.001	8.27 (5.43 – 12.58)	<0.001
**eGFR (ml/min/1.73m^2) [N=1007]**		0.99 (0.98 – 1.00)	0.013	1.01 (1.00 – 1.02)	0.139
**uACR (KDIGO categories) (vs. category A1, <3mg/mmol) [N=1007]**	A2 (3-29 mg/mmol)	1.24 (0.92 - 1.68)	0.090#	0.92 (0.63 – 1.34)	0.767#
	A3 (≥30mg/mmol)	1.72 (0.97 – 3.09)		1.19 (0.59 – 2.43)	
**Educational attainment (vs. first or higher degree or NVQ 4-5) [N=1007]**	No formal qualifications	1.88 (1.35 – 2.62)	<0.001#	1.22 (0.82 – 1.82)	0.549#
	GCSE, A level or NVQ 1-3	1.30 (0.90 – 1.87)		1.24 (0.82 – 1.89)	
**BMI (vs. <25 kg/m^2))**	Overweight (BMI 25-29.99 kg/m^2)	1.54 (1.08 – 2.21)	<0.001#	1.38 (0.92 – 2.06)	0.019#
	Obese (BMI ≥30 kg/m^2)	2.81 (1.96 – 4.03)		1.82 (1.19 – 2.76)	
**Smoking status (vs. never smoked)**	Current smoker	0.85 (0.46 – 1.60)	0.421#	-	-
	Ex-smoker	1.15 (0.89 – 1.49)		-	

*Adjusted for age, deprivation level, number of comorbidities, functional status, eGFR at five-year follow-up, uACR at five-year follow-up, educational attainment, and BMI. #p value for trend. Abbreviations: OR= Odds Ratio, CI= Confidence Interval, KPS= Karnofsky Performance Status, eGFR= estimated Glomerular Filtration Rate, uACR= Urinary Albumin to Creatinine Ratio, KDIGO= Kidney Disease Improving Global Outcomes,

Fraser SDS, *et al. BMJ Open* 2020; 10:e040286. doi: 10.1136/bmjopen-2020-040286

GCSE= General Certificate of Secondary Education, A level= Advanced level, NVQ= National Vocational Qualifications, BMI= Body Mass Index, IMD= Index of Multiple Deprivation.

S4. SELF CARE

		Univariable		Multivariable*	
		OR (95% CI)	p	OR (95% CI)	p
**Age (years)**		1.01 (0.99 – 1.03)	0.472	-	-
**Female sex (vs. male)**		0.91 (0.64 – 1.27)	0.568	-	-
**Index of multiple deprivation (IMD quintile relative to England) (vs. quintile 5: least deprived) [N=1006]**	Quintile 1 (most deprived)	2.27 (1.19 – 4.34)	0.041#	1.12 (0.51 – 2.42)	0.895#
	Quintile 2	1.90 (1.15 – 3.15)		1.20 (0.67 – 2.19)	
	Quintile 3	1.64 (0.95 – 2.84)		1.31 (0.68 – 2.51)	
	Quintile 4	1.26 (0.75 – 2.13)		0.99 (0.54 – 1.85)	
**Number of comorbidities (vs. no comorbidities)**	One	1.43 (0.41 – 4.92)	<0.001#	1.27 (0.34 – 4.84)	0.050#
	Two	3.37 (1.01 – 11.21)		1.89 (0.51 – 6.97)	
	Three or more	6.45 (1.97 – 21.15)		2.64 (0.72 – 9.67)	
**Functional status (KPS score) (vs. KPS >70)**	Functional impairment (KPS ≤70)	15.39 (10.43 – 22.69)	<0.001	13.08 (8.46 – 20.22)	<0.001
**eGFR (ml/min/1.73m^2) [N=1007]**		0.99 (0.98 – 1.00)	0.055	1.01 (0.99 – 1.21)	0.154
**uACR (KDIGO categories) (vs. category A1, <3mg/mmol) [N=1007]**	A2 (3-29 mg/mmol)	1.37 (0.93 - 2.03)	0.223 #	-	-
	A3 (≥30mg/mmol)	1.39 (0.67 – 2.86)		-	
**Educational attainment (vs. first or higher degree or NVQ 4-5) [N=1007]**	No formal qualifications	1.91 (1.18 – 3.08)	0.014#	1.11 (0.63 – 1.97)	0.669#
	GCSE, A level or NVQ 1-3	1.31 (0.76 – 2.23)		1.32 (0.69 – 2.50)	
**BMI (vs. <25 kg/m^2))**	Overweight (BMI 25-29.99 kg/m^2)	1.20 (0.68 – 2.13)	<0.001#	0.95 (0.49 – 1.82)	0.003#
	Obese (BMI ≥30 kg/m^2)	3.47 (2.03 – 5.92)		1.98 (1.06 – 3.71)	
**Smoking status (vs. never smoked)**	Current smoker	2.17 (1.05 – 4.51)	0.018#	2.53 (1.00 – 6.40)	0.006#
	Ex-smoker	1.53 (1.08 – 2.16)		1.89 (1.24 – 2.88)	

* Adjusted for deprivation level, number of comorbidities, functional status, eGFR at five-year follow-up, educational attainment, BMI and smoking. #p value for trend. Abbreviations: OR= Odds Ratio, CI= Confidence Interval, KPS= Karnofsky Performance Status, eGFR= estimated Glomerular Filtration Rate, uACR= Urinary Albumin to Creatinine Ratio, KDIGO= Kidney Disease Improving Global Outcomes, GCSE= General Certificate of Secondary Education, A level= Advanced level, NVQ= National Vocational Qualifications, BMI= Body Mass Index, IMD= Index of Multiple Deprivation.

Fraser SDS, *et al. BMJ Open* 2020; 10:e040286. doi: 10.1136/bmjopen-2020-040286

S5. PAIN / DISCOMFORT

		Univariable		Multivariable*	
		**OR (95% CI)**	**p**	**OR (95% CI)**	**p**
**Age (years)**		1.02 (1.00 – 1.03)	0.026	1.01 (0.99 – 1.02)	0.550
**Female sex (vs. male)**		1.23 (0.93 – 1.63)	0.141	-	-
**Index of multiple deprivation (IMD quintile relative to England) (vs. quintile 5: least deprived) [N=1006]**	Quintile 1 (most deprived)	2.42 (1.30 – 4.50)	0.046[#]	1.87 (0.97 – 3.60)	0.381[#]
	Quintile 2	1.52 (1.03 – 2.24)		1.33 (0.88 – 2.03)	
	Quintile 3	1.27 (0.84 – 1.92)		1.26 (0.81 – 1.95)	
	Quintile 4	1.25 (0.86 – 1.82)		1.24 (0.83 – 1.84)	
**Number of comorbidities (vs. no comorbidities)**	One	1.62 (0.91 – 2.86)	<0.001[#]	1.49 (0.82 – 2.71)	<0.001[#]
	Two	2.95 (1.65 – 5.29)		2.27 (1.22 – 4.20)	
	Three or more	4.70 (2.60 – 8.48)		3.06 (1.63 – 5.73)	
**Functional status (KPS score) (vs. KPS >70)**	Functional impairment (KPS ≤70)	4.29 (2.78 – 6.61)	<0.001	2.94 (1.86 – 4.67)	<0.001
**eGFR (ml/min/1.73m^2) [N=1007]**		0.99 (0.98 – 1.00)	0.150	-	-
**uACR (KDIGO categories) (vs. category A1, <3mg/mmol) [N=1007]**	A2 (3-29 mg/mmol)	0.87 (0.62 - 1.20)	0.670[#]	-	-
	A3 (≥30mg/mmol)	1.04 (0.55 – 1.96)		-	
**Educational attainment (vs. first or higher degree or NVQ 4-5) [N=1007]**	No formal qualifications	1.67 (1.19 – 2.35)	0.007[#]	1.23 (0.84 – 1.97)	0.075[#]
	GCSE, A level or NVQ 1-3	1.65 (1.13 – 2.41)		1.59 (1.06 – 2.38)	
**BMI (vs. <25 kg/m^2))**	Overweight (BMI 25-29.99 kg/m^2)	1.63 (1.15 – 2.30)	<0.001[#]	1.47 (1.02 – 2.12)	<0.001[#]
	Obese (BMI ≥30 kg/m^2)	3.26 (2.23 – 4.76)		2.37 (1.58 – 3.55)	
**Smoking status (vs. never smoked)**	Current smoker	1.12 (0.56 – 2.24)	0.945[#]	-	-
	Ex-smoker	1.02 (0.77 – 1.34)		-	

Adjusted for age, deprivation level, number of comorbidities, functional status, educational attainment, and BMI. [#]p value for trend. Abbreviations: OR= Odds Ratio, CI= Confidence Interval, KPS= Karnofsky Performance Status, eGFR= estimated Glomerular Filtration Rate, uACR= Urinary Albumin to Creatinine Ratio, KDIGO= Kidney Disease Improving Global Outcomes, GCSE= General Certificate of Secondary Education, A level= Advanced level, NVQ= National Vocational Qualifications, BMI= Body Mass Index, IMD= Index of Multiple Deprivation.

Fraser SDS, *et al. BMJ Open* 2020; 10:e040286. doi: 10.1136/bmjopen-2020-040286

S6. ANXIETY / DEPRESSION

		Univariable		Multivariable*	
		**OR (95% CI)**	**p**	**OR (95% CI)**	**p**
**Age (years)**		1.00 (0.98 – 1.01)	0.942	-	-
**Female sex (vs. male)**		1.63 (1.23 – 2.16)	0.001	1.60 (1.18 –2.16)	0.002
**Index of multiple deprivation (IMD quintile relative to England) (vs. quintile 5: least deprived) [N=1006]**	Quintile 1 (most deprived)	0.89 (0.51 – 1.54)	0.856#	-	-
	Quintile 2	0.94 (0.64 – 1.38)		-	
	Quintile 3	1.14 (0.76 – 1.72)		-	
	Quintile 4	0.95 (0.65 – 1.38)		-	
**Number of comorbidities (vs. no comorbidities)**	One	0.80 (0.43 – 1.51)	0.005#	0.94 (0.49 – 1.81)	0.269#
	Two	1.25 (0.67 – 2.34)		1.29 (0.67 – 2.47)	
	Three or more	1.48 (0.80 – 2.75)		1.30 (0.68 – 2.49)	
**Functional status (KPS score) (vs. KPS >70)**	Functional impairment (KPS ≤70)	3.12 (2.30 – 4.22)	<0.001	3.08 (2.23 – 4.27)	<0.001
**eGFR (ml/min/1.73m^2) [N=1007]**		1.00 (0.99 – 1.01)	0.998	-	-
**uACR (KDIGO categories) (vs. category A1, <3mg/mmol) [N=1007]**	A2 (3-29 mg/mmol)	1.00 (0.72 - 1.38)	0.851#	-	-
	A3 (≥30mg/mmol)	0.83 (0.44 – 1.57)		-	
**Educational attainment (vs. first or higher degree or NVQ 4-5) [N=1007]**	No formal qualifications	1.51 (1.04 – 2.18)	0.009#	1.05 (0.71 – 1.56)	0.009#
	GCSE, A level or NVQ 1-3	1.86 (1.25 – 2.77)		1.67 (1.10 – 2.52)	
**BMI (vs. <25 kg/m^2))**	Overweight (BMI 25-29.99 kg/m^2)	0.94 (0.65 – 1.36)	0.133#	-	-
	Obese (BMI ≥30 kg/m^2)	1.26 (0.87 – 1.82)		-	
**Smoking status (vs. never smoked)**	Current smoker	1.00 (0.51 – 1.94)	0.989#	-	-
	Ex-smoker	0.98 (0.75 – 1.29)		-	

* Adjusted for sex, number of comorbidities, and functional status. #p value for trend. Abbreviations: OR= Odds Ratio, CI= Confidence Interval, KPS= Karnofsky Performance Status, eGFR= estimated Glomerular Filtration Rate, uACR= Urinary Albumin to Creatinine Ratio, KDIGO= Kidney Disease Improving Global Outcomes, GCSE= General Certificate of Secondary Education, A level= Advanced level, NVQ= National Vocational Qualifications, BMI= Body Mass Index, IMD= Index of Multiple Deprivation.

Fraser SDS, *et al. BMJ Open* 2020; 10:e040286. doi: 10.1136/bmjopen-2020-040286
