# Supplementary material for: Health-related quality of life, functional impairment and comorbidity in people with mild-to-moderate chronic kidney disease: a cross-sectional study
Source: BMJ Open. 2020 Aug 6;10(8):e040286. doi: 10.1136/bmjopen-2020-040286 (PMC7412591; doi:10.1136/bmjopen-2020-040286)
Supplement: Author's manuscript [file bmjopen-2020-040286.draft_revisions.pdf]

BMJ Open

BMJ Open is committed to open peer review. As part of this commitment we make the peer review history of every article we publish publicly available.

When an article is published we post the peer reviewers' comments and the authors' responses online. We also post the versions of the paper that were used during peer review. These are the versions that the peer review comments apply to.

The versions of the paper that follow are the versions that were submitted during the peer review process. They are not the versions of record or the final published versions. They should not be cited or distributed as the published version of this manuscript.

BMJ Open is an open access journal and the full, final, typeset and author-corrected version of record of the manuscript is available on our site with no access controls, subscription charges or pay-per-view fees (http://bmjopen.bmj.com).

If you have any questions on BMJ Open's open peer review process please email info.bmjopen@bmj.com

BMJ Open

Health related quality of life, functional impairment and comorbidity in people with mild to moderate chronic kidney disease: a cross sectional study

Journal:	*BMJ Open*
Manuscript ID	bmjopen-2020-040286
Article Type:	Original research
Date Submitted by the Author:	09-May-2020
Complete List of Authors:	Fraser, Simon; University of Southampton, Faculty of Medicine<br>Barker, Jenny; University of Southampton, Faculty of Medicine<br>Roderick, Paul; University of Southampton, Faculty of Medicine<br>Yuen, Ho Ming; University of Southampton, Faculty of Medicine<br>Shardlow, Adam; Royal Derby Hospital, Renal Medicine<br>Morris, James; University of Southampton, Faculty of Medicine<br>McIntyre, Natasha; Royal Derby Hospital, Renal Medicine<br>Fluck, Richard; Royal Derby Hospital, Renal Medicine<br>McIntyre, CW; University of Western Ontario, Division of Nephrology<br>Taal, Maarten; Royal Derby Hospital, Renal Medicine; University of Nottingham, Centre for Kidney Research and Innovation
Keywords:	Chronic renal failure < NEPHROLOGY, EPIDEMIOLOGY, PRIMARY CARE, PUBLIC HEALTH

SCHOLARONE™
Manuscripts

I, the Submitting Author has the right to grant and does grant on behalf of all authors of the Work (as defined in the below author licence), an exclusive licence and/or a non-exclusive licence for contributions from authors who are: i) UK Crown employees; ii) where BMJ has agreed a CC-BY licence shall apply, and/or iii) in accordance with the terms applicable for US Federal Government officers or employees acting as part of their official duties; on a worldwide, perpetual, irrevocable, royalty-free basis to BMJ Publishing Group Ltd ("BMJ") its licensees and where the relevant Journal is co-owned by BMJ to the co-owners of the Journal, to publish the Work in this journal and any other BMJ products and to exploit all rights, as set out in our licence.

The Submitting Author accepts and understands that any supply made under these terms is made by BMJ to the Submitting Author unless you are acting as an employee on behalf of your employer or a postgraduate student of an affiliated institution which is paying any applicable article publishing charge ("APC") for Open Access articles. Where the Submitting Author wishes to make the Work available on an Open Access basis (and intends to pay the relevant APC), the terms of reuse of such Open Access shall be governed by a Creative Commons licence – details of these licences and which Creative Commons licence will apply to this Work are set out in our licence referred to above.

Other than as permitted in any relevant BMJ Author's Self Archiving Policies, I confirm this Work has not been accepted for publication elsewhere, is not being considered for publication elsewhere and does not duplicate material already published. I confirm all authors consent to publication of this Work and authorise the granting of this licence.

For peer review only

Health related quality of life, functional impairment and comorbidity in people with mild to moderate chronic kidney disease: a cross sectional study

Corresponding author:

Simon DS Fraser[1], School of Primary Care, Population Sciences and Medical Education, Faculty of Medicine, University of Southampton, South Academic Block, Southampton General Hospital, Tremona Road, Southampton, Hampshire, UK, SO16 6YD. email: s.fraser@soton.ac.uk

Co-authors:

Jenny Barker[1]

Paul J Roderick[1]

Ho Ming Yuen[1]

Adam Shardlow[2]

James E Morris[1]

Natasha J McIntyre[2]

Richard J Fluck[3]

Chris W McIntyre[4]

Maarten W Taal[2]

Institutions:

1. School of Primary Care, Population Sciences and Medical Education, Faculty of Medicine, University of Southampton, South Academic Block, Southampton General Hospital, Tremona Road, Southampton, Hampshire, UK, SO16 6YD.
2. Division of Medical Sciences and Graduate-Entry Medicine, University of Nottingham, Derby, UK
3. The Department of Renal Medicine, Royal Derby Hospital NHS Foundation Trust, Derby, Derbyshire, UK
4. Department of Medical Biophysics, Medical Sciences Building, Western University, London, Ontario, Canada

Word count (excluding title page, abstract, references, figures and tables): **3666**

For peer review only

For peer review only - http://bmjopen.bmj.com/site/about/guidelines.xhtml

Abstract

Objectives
To determine the associations between comorbidities, health related quality of life (HRQoL) and functional impairment in people with mild to moderate chronic kidney disease (CKD) in primary care.

Design
Cross-sectional analysis at five-year follow-up in a prospective cohort study

Setting
Thirty- two general practitioner (GP) surgeries in England

Participants
1008 participants with CKD stage 3 (of 1741people recruited at baseline in the Renal Risk in Derby study) who survived to five years and had complete follow-up data for HRQoL and functional status (FS).

Primary and secondary outcome measures
HRQoL assessed using the EQ-5D-5L (using EQ-5D-5L domains of mobility, self care, usual activities, pain/discomfort and anxiety/depression and index value using utility scores calculated from the English general population), and FS using the Karnofsky Performance Status scale (functional impairment defined as Karnofksy score ≤70). Comorbidity was defined by self-reported or doctor-diagnosed condition, disease-specific medication or blood result.

Results
Mean age was 75.8 years. The numbers reporting some problems in EQ-5D-5L domains were: 582 (57.7%) for mobility, 166 (16.5%) for self-care, 466 (46.2%) for usual activities, 712 (70.6%) for pain/discomfort, and 319 (31.6%) for anxiety/depression. Only 191 (18.9%) reported no problems in any domain. HRQoL index values showed greater variation among those with lower FS. 234 (23.2%) had functional impairment.
In multivariable logistic regression models functional impairment was independently associated with experiencing problems for all EQ-5D-5L domains. Higher comorbidity count and obesity were independently associated with problems in mobility, self-care, usual activities and pain/discomfort. Female sex, lower FS and lower educational attainment were independently associated with anxiety/depression. Older age, higher comorbidity count, albuminuria, lower educational attainment and obesity were independently associated with functional impairment.

Conclusions
The majority of persons with mild to moderate CKD reported reductions in at least one HRQoL domain, which were independently associated with comorbidities, obesity and functional impairment.

Trial registration number
National Institute for Health Research Clinical Research Portfolio Study Number 6632

For peer review only - http://bmjopen.bmj.com/site/about/guidelines.xhtml

Strengths and limitations of this study

- This study involved a large cohort of people with CKD recruited from primary care, a setting in which patients with mild to moderate CKD are typically managed in the UK.

- A broad range of comorbidities were included but they were identified at baseline only, not at follow up, by which time the number of comorbidities may have changed.

- Health related quality of life and functional status were measured in the same patient group and the use of the EQ-5D-5L index measure and data from the HSE enabled comparison with a general population.

- Health related quality of life and functional status measures were taken at five-year follow-up but not at baseline and we were therefore unable to identify change over time.

- This was a cross-sectional study of survivors and we are therefore not able to draw causative links.

For peer review only

INTRODUCTION

Chronic kidney disease (CKD) is common globally, affecting about 13% of the general adult population, with CKD stage 3 the most prevalent category.[1,2] Current treatment guidelines for CKD are disease-specific and focus on reducing progression and preventing complications such as cardiovascular disease.[3] However, in the UK most people with CKD stage 3 are managed in primary care and in this context only a minority (18%) evidence progression over 5 years.[4] The risk of end-stage kidney disease (ESKD) is extremely low (0.2%).[4]

Conversely, comorbidities (additional chronic diseases) are common in individuals with CKD and can worsen clinical outcomes and health related quality of life (HRQoL).[5] 96% of people with stage 3 disease have at least one comorbidity, around 40% have a comorbidity count of two or more.[6]

A significant body of research has explored HRQoL and the functional status (FS) of people with ESKD or following kidney transplant but these factors are not well explored in those with less severe CKD. Among 733 people with high risk CKD in the Renal Impairment In Secondary Care (RIISC) study, 555 (76%) reported problems in one or more of the EQ5D domains.[7,8]

This is a clinically important knowledge gap because mild to moderate reductions in glomerular filtration rate (GFR) are usually asymptomatic, so improved understanding of the comorbidities and symptoms that affect HRQoL and FS in this group of people is important to facilitate a holistic approach to management. The objective of this study was therefore to evaluate HRQoL and determine the associations between comorbidities, HRQoL and functional impairment in people with mild to moderate CKD in primary care.

MATERIALS AND METHODS

A detailed description of the Renal Risk in Derby (RRID) study methodology has been published elsewhere.[9] In summary, approximately 8,280 people with CKD stage 3 were identified from renal registers at 32 primary care clinics in Derbyshire, UK between 2008 and 2010 and invited to participate in the study. Of these people, 1,822 attended initial baseline visits and 1,741 met eligibility criteria (age ≥ 18 years; two estimated GFR (eGFR) results (derived from the Modification of Diet in Renal Disease study [MDRD] equation) of 30–59 ml/min/1.73 m^2, at least 90 days apart).[9] People with a life expectancy of less than one year, who were unable to attend study visits, or who had a solid organ transplant were excluded.

Health related quality of life

HRQoL was assessed at five-year follow-up using the EQ-5D-5L, a widely used, validated measure of health status that can be standardised to different populations. EQ-5D-5L consists of two aspects: a descriptive system, in which participants are asked to rate their health state from 1-5 against five domains (mobility, self-care, usual activities, pain/discomfort, anxiety/depression) and the EQ visual analogue scale (EQ-VAS), in which participants rate their health on a scale ranging from 'the best health you can imagine,' (100) to 'the worst health you can imagine,' (0).[10] An EQ-5D-5L value set has previously been published for England.[11] However, concerns have been raised about the quality and reliability of the data collected in the valuation study such that the English National Institute for Health and Care Excellence (NICE) recommend 'If data were gathered using the EQ-5D-5L descriptive system, utility values in reference-case analyses should be calculated by mapping the 5L descriptive system data onto the 3L value set'. [12] For these analyses, individual health states were therefore converted using the Euroqol EQ-5D-5L Crosswalk Index Value Calculator into a single 3L index value (a preference-based score that typically ranges from states worse than dead (<0) to 1 (full health) with dead at 0) using utility scores calculated from the English general

population.[13,14] The index value and the EQ-VAS score were used to graphically display the relationship between HRQoL and FS.

Functional status

FS, defined in this paper as the physical ability to perform normal activities and independently self-care, was assessed at five-year follow-up using the Karnofsky Performance Status (KPS) scale. The KPS is a clinician-assessed score originally developed in oncology and was used for assessing prognosis and management in cancer patients.[15] The scale ranges from 'normal/ no complaints,' (100) to 'dead' (0). Theoretically the scale can take any whole number value within the range, but in practice results are commonly recorded as multiples of ten, therefore KPS was treated as an ordinal variable in this study. The original continuous KPS score >70 is defined as being 'able to carry on normal activity and to work with no special care needed,' a score of between 50 and 70 inclusive is defined as 'unable to work, able to live at home and care for most personal needs; varying amount of assistance needed', and a score of less than or equal to 40 is defined as 'unable to care for self, requiring the equivalent of institutional or hospital care'.[15] Functional impairment was analysed as a binary outcome due to the small number of patients with low KPS score. A KPS score of ≤ 70 vs. > 70 was chosen to compare those able to carry on normal life with those experiencing some functional impairment as has been used in evaluation of FS in patients with lung cancer.[16]

Comorbidities identified at baseline

The methods for defining comorbidities in participants have been described in detail elsewhere.[6] In brief, eleven comorbidities were pragmatically identified at baseline using information from a combination of sources and agreed by consensus between three clinicians (SF, MWT and PJR): patient questionnaires in which patients were asked to list chronic medications (followed by verbal confirmation with verification of repeat prescriptions where possible), blood pressure measurement at the time of baseline study visit, and self-reported clinical diagnoses. Self-reported comorbidities included heart failure, ischaemic heart disease, peripheral vascular disease (defined as peripheral arterial revascularization or amputation) and cerebrovascular disease (stroke or transient ischaemic attack). Diagnoses of chronic respiratory disorder, depression, painful condition, hypertension, diabetes and thyroid disorders were made according to medication history or patient report. Anaemia was defined according to Kidney Disease Improving Global Outcomes (KDIGO) guidelines as haemoglobin <13.0 g/dl (<130 g/l) in males and <12.0 g/dl (<120 g/l) in females, at baseline.[17] Hypertension was defined either by medication history, or by a systolic blood pressure >140mmHg or diastolic >90mmHg, at baseline.

Kidney function

Kidney function was assessed at five-year follow-up. eGFR was calculated using the Chronic Kidney Disease Epidemiology Collaboration (CKDEPI) equation and was treated as a continuous variable. The urine albumin-to-creatinine ratio (uACR) from three consecutive early morning specimens was used for analysis. uACR was categorised into three levels according to KDIGO guidelines and fitted as a discrete variable in regression analyses.

Methods for defining CKD progression have also been detailed elsewhere.[4] In summary, progression of CKD was defined as a 25% decline in GFR, coupled with a worsening of GFR category or an increase in albuminuria category. CKD remission was defined as the presence of both eGFR >60 ml/min/1.73 m^2 and uACR <3 mg/mmol at any study visit in an individual who had previously met KDIGO diagnostic criteria for CKD.

Other baseline measures

Body mass index (BMI) was calculated from weight in kilograms divided by square of height in metres and was treated as a categorical variable.[18] Smoking status was categorized as never

For peer review only - http://bmjopen.bmj.com/site/about/guidelines.xhtml

smoked, ex-smoker, and current smoker. Socioeconomic status was assessed using self-reported educational attainment (categorized into no formal qualifications, school or equivalent qualifications, and degree or equivalent qualifications) as well as the Index of Multiple Deprivation (IMD) score, categorized in quintiles.[19] The IMD is a measure of relative deprivation for small areas of residence in England and combines information from seven domains: income; employment; education, skills and training; health and disability; crime; barriers to housing and services; and living environment. Self-reported ethnicity status was also collected.

Statistical analyses

Descriptive statistics were used to show the characteristics of the study participants at five-year follow-up. Descriptive statistics were also used to show the distribution of functional impairment (KPS ≤70) among those reporting problems in the five EQ-5D-5L domains. Associations between the patient-reported EQ-5D-5L domains and FS was assessed using the Chi-square test. Ratings from the five participant-reported EQ-5D-5L domains were also compared between the RRID cohort and those reported by people aged 65 years and over in the 2012 Health Survey for England (HSE) – which is representative of the England population.[20] A comparison of basic characteristics was also made between those with and without complete five year follow up data.

Univariable logistic regression models were used to assess the relationships between having 'some problems' in each EQ-5D-5L domain and each predictor variable, including comorbidity count and year-five eGFR. Variables considered to be clinically relevant and where p<0.1 on univariable analysis were subsequently included in multivariable logistic regression models. This process was then repeated for the relationship with the outcome variable functional impairment. Due to the small number of non-white participants, ethnicity was not included in the models.

In the regression models, interactions between the individual and area measures of socioeconomic status were also tested because of the potential for the relationship between individual socioeconomic status (indicated by educational attainment) and HRQoL to vary by area deprivation, particularly for older people.[21] The level of significance was set at 5%. All analyses were performed using Stata/IC version 15.0.

The RRID study was approved by the Nottingham Research Ethics Committee 1 and was included on the National Institute for Health Research (NIHR) Clinical Research Portfolio (NIHR Study ID: 6632). All participants provided written, informed consent. The RRID study complies with the Declaration of Helsinki and the principles of Good Clinical Practice.

Patient and public involvement

The RRID study design was discussed with a patient and two feedback meetings for participants and their families were organised after the year five visits, which were well attended. In addition, a web page provides updates and information for participants: https://www.uhdb.nhs.uk/renal-risk-in-derby-rrid-study/

RESULTS

Of 1741 participants recruited, 1494 survived to five years, and of these 1008 participants (67% of survivors) had complete five-year follow-up data for HRQoL and FS (Figure 1). The mean age of the cohort was 75.8 years (SD 8.6) and the majority (n=621, 61.6%) were female (Table 1).

Table 1. Characteristics of patients at five-year follow-up in the Renal Risk in Derby study, N=1008 unless otherwise stated

**Variable**	**Category**	**Descriptive statistics**
**Age in years**[#], mean (SD)		75.8 (8.6)

For peer review only - http://bmjopen.bmj.com/site/about/guidelines.xhtml

**Age group**#, n (%)	<70 years	220 (21.8)
	70-80 years	467 (46.3)
	>80 years	321 (31.8)
**Sex***, n (%)	Male	387 (38.4)
	Female	621 (61.6)
**Ethnicity***, n (%)	White	994 (98.6)
	Other**	14 (1.4)
**Educational attainment***, n (%) [N=1007]	No formal qualifications	506 (50.3)
	GCSE, A level, NVQ 1-3	291 (28.9)
	First or higher degree, NVQ 4-5	210 (20.9)
**Index of multiple deprivation** (IMD quintile relative to England)*, n (%) [N=1006]	Quintile 1 (most deprived)	82 (8.2)
	Quintile 2	243 (24.2)
	Quintile 3	184 (18.3)
	Quintile 4	258 (25.6)
	Quintile 5 (least deprived)	239 (23.8)
**Body mass index***, n (%)	Normal or underweight (<25 kg/m^2)	195 (19.3)
	Overweight (25-29.99 kg/m^2)	422 (41.9)
	Obese (≥30 kg/m^2)	391 (38.8)
**Smoking status***, n (%)	Never smoked	496 (49.2)
	Ex-smoker	468 (46.4)
	Current smoker	44 (4.4)
**eGFR# in mL/min/1.73m^2**, mean (SD) [N=1007]		54.0 (15.2)
**uACR# in mg/mmol,** median (IQR) [N=1007]		0.7 (0-3.3)
**KDIGO uACR categories#** n (%)	A1	741 (73.5)
	A2	217(21.5)
	A3	50 (5.0)
**KDIGO eGFR categories#** (eGFR in mL/min/1.73m^2)	G1 (eGFR ≥90)	13 (1.3)
	G2 (eGFR 60-89)	337 (33.4)
	G3a (eGFR 45-59)	379 (37.6)
	G3b (eGFR 30-44)	223 (22.1)
	G4 (eGFR 15-29)	55 (5.5)
	G5 (eGFR <15)	1 (0.1)
**Progression of kidney disease#**, n (%)	Stable	460 (45.6)
	Progression	244 (24.2)
	Remission	304 (30.2)
**Number of comorbidities***, n (%)	None (CKD only)	56 (5.6)
	One	308 (30.6)
	Two	300 (29.8)
	Three or more	344 (34.1)
**Individual comorbidities***, n (%)	Hypertension	874 (86.7)
	Painful condition	300 (29.8)
	Anaemia	201 (19.9)
	Ischaemic heart disease	187 (18.6)
	Diabetes	143 (14.2)
	Thyroid disorder	127 (12.6)
	Cerebrovascular disease	96 (9.5)
	Chronic respiratory disorder	79 (7.8)
	Depression	59 (5.9)
	Peripheral vascular disease	29 (2.9)
	Heart failure	24 (2.4)
**Quality of Life domains** (Any	Mobility problems	582 (57.7)

For peer review only

problems reported in each EQ-5D-5L domain, n (%))	Self-care problems	166 (16.5)
	Usual activity problems	466 (46.2)
	Pain/discomfort	712 (70.6)
	Anxiety/depression	319 (31.6)
	No problems in any domain	191 (18.9)
**Functional status**# (KPS score), n (%)	Functional impairment (KPS ≤70)	234 (23.2)
	KPS >70 (able to carry on normal activity and to work; no special care needed)	774 (76.8)

* Variables assessed at baseline. # Variables assessed at year five follow up.
** Includes Mixed, Asian, Cypriot and Other. Abbreviations SD= standard deviation, GCSE= General Certificate of Secondary Education, A level= Advanced level, NVQ= National Vocational Qualifications, IMD= Index of Multiple Deprivation, eGFR= estimated Glomerular Filtration Rate, uACR= Urine Albumin-to-Creatinine Ratio, IQR= Interquartile Range, KDIGO= Kidney Disease: Improving Global Outcomes, CKD= Chronic Kidney Disease, KPS= Karnofsky Performance Status

For peer review only

Approximately half (n=506, 50.3%) reported having had no formal education, just under half (n=497, 49.4%) lived in areas of lower deprivation (IMD quintiles four or five) and the majority (n=994, 98.6%) were white. The mean eGFR at follow-up was 54.0 ml/min/1.73m^2 (SD 15.2) and almost half (n=460, 45.6%) had had stable CKD over the preceding five-year period. Only 56 (5.6%) had no comorbidities and about a third (n=344, 34.1%) had three or more comorbidities. For comparison of basic characteristics of those with and without complete five year follow up data see supplementary Table S1. A slightly higher proportion of those with incomplete follow up data had three or more comorbidities and only a very small proportion had functional impairment (Table S1). The majority reported some impairment in HRQoL overall, with a median score of 75 out of 100 (interquartile range 60-90) on the EQ-VAS. A minority (n=378, 37.5%) had an EQ-5D-3L index score higher than the age/sex matched median, and only 18.9% of people (n=191) reported no problems across any of the individual HRQoL domains. Furthermore, a majority of participants reported some problems with mobility (n=582, 57.7%) and pain/discomfort (n=712, 70.6%) (Table 1).

When comparing the self-reported HRQoL domains with HSE data, the proportion of people in the RRID population reporting problems with mobility or pain/discomfort was higher (57.7% vs. 50.4%, and 70.6% vs. 60.1% respectively) than in the HSE population (Table 2).

Table 2. Comparison of the EQ-5D-5L quality of life domains between the Health Survey for England (HSE) 2012 and the Renal Risk in Derby (RRID) cohort

		HSE 2012 cohort* (N=258)		RRID CKD cohort (N=1008)	
		n	%	n	%
**Mobility**	1 (no problems in walking about)	128	49.6	426	42.3
	2 – 5 (some problems)	130	50.4	582	57.7
**Self-care**	1 (no problems washing or dressing)	209	81.0	842	83.5
	2 – 5 (some problems)	49	19.0	166	16.5
**Usual**	1 (no problems doing usual activities)	142	55.0	542	53.8

For peer review only - http://bmjopen.bmj.com/site/about/guidelines.xhtml

**activities**	2 – 5 (some problems)	116	45.0	466	46.2
**Pain/ discomfort**	1 (no pain or discomfort)	103	39.9	296	29.4
	2 – 5 (some pain or discomfort)	155	60.1	712	70.6
**Anxiety/ depression**	1 (not anxious or depressed)	188	72.9	689	68.4
	2 – 5 (some anxiety or depression)	70	27.1	319	31.6

* All participants were aged 65 years or above. CKD= Chronic Kidney Disease

For clinician-assessed FS, only two participants had performance status assessed as KPS ≤40 (‘unable to care for self, requiring the equivalent of institutional or hospice care’) and 232 (23%) were assessed as KPS 50-70 (‘unable to work; able to live at home and care for most personal needs; varying amount of assistance needed’).

The association between clinician-assessed FS and patient-reported HRQoL was complex, either when based on the index score (Figure 2a) or the VAS scale (Figure 2b). HRQoL was generally higher among those with better FS. However, the spread of HRQoL scores (using either of the HRQoL metrics) was broader among those with lower FS, suggesting a greater degree of variation in HRQoL among those with lower FS than among those with higher FS (Figure 2). A higher proportion of people with clinician-assessed functional impairment (KPS ≤70) reported having some degree of problems in each of the five EQ-5D-5L domains than people without functional impairment (Supplementary table S2).

Using the mobility domain as an example (Table 3), on univariable analysis older age, greater area deprivation level, higher number of comorbidities, poorer functional status, lower eGFR, higher level of albuminuria, lower educational attainment, and higher BMI were associated with having some problems.

Table 3. Logistic regression models examining associations between lower quality of life (EQ-5D-5L mobility domain categorised as ‘no problems’ vs. ‘any problems’) and patient characteristics. N=1008 in univariable models unless otherwise stated, N=1005 for final multivariable logistic regression models

		**Univariable**		**Multivariable***	
		**OR (95% CI)**	**p**	**OR (95% CI)**	**p**
**Age (years)**		1.05 (1.03 – 1.07)	<0.001	1.03 (1.02 – 1.05)	0.001
**Female sex (vs. male)**		1.16 (0.89 – 1.49)	0.27	-	-
**Index of multiple deprivation (IMD quintile relative to England) (vs. quintile 5: least deprived)** [N=1006]	Quintile 1 (most deprived)	1.61 (0.96 – 2.69)	0.04#	0.95 (0.52 – 1.74)	0.436#
	Quintile 2	1.69 (1.17 – 2.43)		1.38 (0.90 – 2.10)	
	Quintile 3	1.10 (0.75 – 1.62)		0.94 (0.60 – 1.48)	
	Quintile 4	1.25 (0.88 – 1.78)		1.17 (0.78 – 1.76)	
**Number of comorbidities (vs. no comorbidities)**	One	1.25 (0.70 – 2.24)	<0.001#	1.01 (0.53 – 1.93)	0.002#
	Two	2.43 (1.35 – 4.38)		1.40 (0.72 – 2.71)	
	Three or more	4.50 (2.49 – 8.12)		2.10 (1.08 – 4.10)	
**Functional status (KPS score) (vs. KPS >70)**	Functional impairment (KPS ≤70)	26.03 (13.60 – 49.81)	<0.001	16.87 (8.70 – 32.79)	<0.001
**eGFR (ml/min/1.73m^2)**		0.98 (0.98 – 0.99)	<0.001	0.99 (0.99 – 1.01)	0.983

For peer review only - http://bmjopen.bmj.com/site/about/guidelines.xhtml

[N=1007]					
**uACR (KDIGO categories) (vs. category A1, <3mg/mmol)** [N=1007]	A2 (3-29 mg/mmol)	1.39 (1.02 - 1.91)	0.074#	1.01 (0.69 – 1.48)	0.937#
	A3 (≥30mg/mmol)	1.42 (0.78 – 2.57)		0.88 (0.41 – 1.85)	
**Educational attainment (vs. first or higher degree or NVQ 4-5)** [N=1007]	No formal qualifications	2.20 (1.59 – 3.05)	<0.001#	1.38 (0.93 – 2.04)	0.238#
	GCSE, A level or NVQ 1-3	1.23 (0.86 – 1.76)		1.13 (0.75 – 1.70)	
**BMI (vs. <25 kg/m²))**	Overweight (BMI 25-29.99 kg/m²)	1.51 (1.07 – 2.13)	<0.001#	1.37 (0.93 – 2.01)	<0.001#
	Obese (BMI ≥30 kg/m²)	3.24 (2.26 – 4.63)		2.44 (1.61 – 3.69)	
**Smoking status (vs. never smoked)**	Current smoker	1.50 (0.79 – 2.87)	0.413#	-	-
	Ex-smoker	1.10 (0.85 – 1.42)		-	

* Adjusted for age, deprivation level, number of comorbidities, functional status, eGFR at five-year follow-up, uACR at five-year follow-up, educational attainment, and BMI. #p value for trend. Abbreviations: OR= Odds Ratio, CI= Confidence Interval, KPS= Karnofsky Performance Status, eGFR= estimated Glomerular Filtration Rate, uACR= Urinary Albumin to Creatinine Ratio, KDIGO= Kidney Disease Improving Global Outcomes, GCSE= General Certificate of Secondary Education, A level= Advanced level, NVQ= National Vocational Qualifications, BMI= Body Mass Index, IMD= Index of Multiple Deprivation.

In the fully adjusted multivariable model these associations remained for older age, higher number of comorbidities, poorer functional status, and higher BMI (Table 3). A summary of the main independent associations identified in the multivariable logistic regression models for usual activities, self-care, pain/discomfort and anxiety/depression are shown in Table 4 and the full analyses in Supplementary tables S3 to S6.

Table 4. Summary matrix of the <u>independent</u> associations with 'some problems' in each domain of the EQ-5D-5L (from multivariable logistic regression analyses)

	Mobility	Self care	Usual activities	Pain / discomfort	Anxiety / depression
**Increasing age**	○				
**Female sex**					○
**Greater area deprivation level**					
**Higher number of comorbidities**	○	○	○	○	

For peer review only

For peer review only - http://bmjopen.bmj.com/site/about/guidelines.xhtml

**Functional impairment**	○	○	○	○	○
**Lower eGFR**					
**Higher level of albuminuria**					
**Lower educational attainment**					○
**Higher BMI**	○	○	○	○	
**Smoking**		○			

Factors associated with a lower FS on univariable analysis included older age, lower socioeconomic status (assessed by both IMD score and educational attainment), higher number of comorbidities, obesity, reduced eGFR and greater degree of albuminuria. Other than reduced eGFR, all of these factors remained significant after adjustment (Table 5). No interactions were identified in any analyses.

For peer review only

Table 5. Logistic regression models of the associations between clinician-assessed functional impairment (KPS score ≤70) and patient characteristics. N=1008 in univariable models unless otherwise stated, N=1004 for final multivariable logistic regression model

		**Univariable**		**Multivariable***	
		**OR (95% CI)**	**p**	**OR (95% CI)**	**p**
**Age (years)**		1.07 (1.05 – 1.09)	<0.001	1.07 (1.04 – 1.09)	<0.001
**Sex (vs. male)**		1.15 (0.85 – 1.56)	0.371	1.32 (0.91 – 1.91)	0.148
**Index of multiple deprivation** (IMD quintile relative to England) **(vs. quintile 5: least deprived)** [N=1006]	Quintile 1 (most deprived)	2.77 (1.55 – 4.95)	0.003#	2.03 (1.06 – 3.87)	0.045#
	Quintile 2	2.19 (1.39 – 3.44)		2.05 (1.24 – 3.40)	
	Quintile 3	1.83 (1.12 – 2.98)		2.02 (1.17 – 3.47)	
	Quintile 4	1.64 (1.03 – 2.59)		1.65 (1.00 – 2.75)	
**Number of comorbidities (vs. no comorbidities)**	One	1.18 (0.44 – 3.18)	<0.001#	0.62 (0.22 – 1.77)	<0.001#
	Two	3.10 (1.19 – 8.08)		1.22 (0.44 – 3.38)	
	Three or more	5.97 (2.32 – 15.35)		2.18 (0.80 – 5.96)	
**eGFR (ml/min/1.73m^2)** [N=1007]		0.97 (0.96 – 0.98)	<0.001	0.99 (0.98 – 1.00)	0.186
**uACR (KDIGO categories) (vs. category A1, <3mg/mmol)** [N=1007]	A2 (3-29mg/mmol)	1.92 (1.37 - 2.70)	0.002#	1.92 (1.29 – 2.87)	0.005#
	A3 (≥30mg/mmol)	1.96 (1.05 – 3.66)		1.74 (0.82 – 3.68)	
**Educational attainment (vs. first or higher degree or NVQ 4-5)** [N=1007]	No formal qualifications	2.77 (1.81 – 4.26)	<0.001#	2.08 (1.26 – 3.41)	<0.001#
	GCSE, A level or NVQ 1-3	1.07 (0.65 – 1.77)		0.99 (0.56 – 1.75)	
**BMI (vs. <25 kg/m^2)**	Overweight (BMI 25- 29.99 kg/m^2)	1.54 (0.94 – 2.53)	<0.001#	1.59 (0.93 – 2.73)	<0.001#
	Obese (BMI ≥30kg/m^2)	3.76 (2.34 – 6.04)		4.23 (2.48 – 7.20)	
**Smoking status (vs. never smoked)**	Current smoker	1.37 (0.70 – 2.71)	0.563#	-	-
	Ex-smoker	0.95 (0.70 – 1.28)		-	

* Adjusted for age, sex, deprivation level, number of comorbidities, eGFR at five-year follow-up, ACR, educational attainment and BMI. #P value for trend. Abbreviations: OR= Odds Ratio, CI= Confidence Interval, eGFR= Estimated Glomerular Filtration Rate, ACR= Albumin Creatinine Ratio, KDIGO= Kidney Disease Improving Global Outcomes, GCSE= General Certificate of Secondary Education, A level= Advanced level, NVQ= National Vocational Qualifications, BMI= Body Mass Index, IMD= Index of Multiple Deprivation.

For peer review only

For peer review only - http://bmjopen.bmj.com/site/about/guidelines.xhtml

DISCUSSION

In this cross-sectional study of people with mild to moderate CKD who survived to year five in a UK primary care cohort, overall patient-reported HRQoL was relatively high though a substantial proportion of participants reported problems in each HRQoL domain. A majority reported problems with mobility and pain/discomfort. Although most people had a clinician-assessed FS suggesting that they were able to carry on normal activity and to work with no special care needed, about a quarter were assessed as having functional impairment (being unable to work but able to live at home and care for most personal needs with a varying amount of assistance needed). HRQoL was generally higher among those with better FS but there was more variation in HRQoL among those with lower FS, and low FS was independently strongly associated with low HRQoL in regression analyses. Higher number of comorbidities and obesity were independently associated with problems in most EQ-5D-5L domains and with functional impairment. Functional impairment was independently associated with experiencing some problems across all EQ-5D-5L domains.

This study had several strengths, including the large size of the cohort, and recruitment from primary care, a setting in which patients with mild to moderate CKD are typically managed. The RRID cohort is pragmatic and likely to represent a population of typical patients with mild to moderate CKD in the UK.[22,23] We were able to identify a broad range of comorbidities but they were identified at baseline only. The number of comorbidities may therefore have changed by the time of follow-up assessment, meaning that our comorbidity prevalence data were likely underestimates of the true prevalence in some patients. Similarly certain other exposures were assessed at baseline and could potentially have changed by the time of follow-up. We recognise these as important limitations but consider that they are unlikely to significantly alter the main findings of our study with regard to HRQoL and FS. A further strength is that we were able to measure HRQoL and FS in the same patient group and the HRQoL and FS data were relatively complete. The use of the EQ-5D-5L index measure and data from the HSE enabled comparison with a general population. However, the index values for HRQoL required conversion to 3L values as reliable 5L index values are not yet available for all standard populations. Evidence from a previous RRID analysis on prior renal function change provided depth to our analyses for this cross-sectional study. However, there were also several important limitations – this was a cross-sectional study of survivors and we were therefore not able to draw causative links. HRQoL and FS measures were taken at five-year follow-up but not at baseline and we were therefore unable to identify change over time. The RRID cohort is predominantly comprised of people of white ethnicity, limiting generalisability of our findings. Comparison with HSE data was undertaken only via univariable analyses, such that potential confounding factors may have influenced the differences observed between the two groups. We also did not have sufficient numbers to allow for reliable exploration of associations between specific comorbidities and HRQoL or FS. We also recognise the need for caution in the interpretation of the associations between functional impairment and problems in individual domains due to small numbers in some individual categories (leading to wide confidence intervals). A further limitation is that one inclusion criterion was the ability to attend study visits, which would have resulted in some selection bias by excluding the very frail.

People with CKD are likely to have multiple comorbidities due both to the nature of the disease process and the relationship between CKD and older age. We have identified that comorbidity count was an independent determinant of both HRQoL and FS, highlighting the importance of a holistic approach that includes attention to comorbidities in the management of people with mild to moderate CKD. As reported previously, 40% of people in this cohort with stage 3 disease had at least two comorbidities.[6] There are clearly shared risk factors for several of the included comorbidities. It is therefore perhaps unsurprising that in a large cohort of over half a million Canadian patients with

For peer review only - http://bmjopen.bmj.com/site/about/guidelines.xhtml

CKD, comorbidities such as hypertension and diabetes were common (46.6% and 17.8% respectively). However, a substantial number of patients also had 'discordant' comorbidities such as chronic pulmonary disease (14.0%) and 10.6% of patients had chronic pain.[5] Comorbidities were all associated with an increased risk of hospitalisation.[5] It is striking that the majority of people in our cohort reported problems with mobility and chronic pain/discomfort, and that both were more prevalent than in a nationally-representative sample of the English general population of similar age. About 30% of our cohort were taking analgesic medication, but about 71% reported pain or discomfort in the EQ-5D-5L. This likely reflects the association of CKD with comorbidities, since mild to moderate reductions in GFR are unlikely to cause poor mobility or pain. Nevertheless, this observation further highlights the need to pay attention to mobility issues and pain management in order to improve quality of life in people with stage 3 CKD.

Mental health problems are common with 26% of adults in England reporting a diagnosis at some point in time.[24] In our study, about 6% of people were classified as having a depressive condition, defined pragmatically based on current antidepressant use or patient self-report, although about 32% reported some anxiety or depression in the EQ-5D-5L, so this was probably an underestimate. In a large meta-analysis, approximately 25% of adults with CKD stage 1-5 had symptoms suggestive of depression.[25] This appears to persist even in milder forms of the disease and a large study from 2012 showed the prevalence of depression in people with CKD whose eGFR was ≥60 ml/min/1.73m^2 was 23.6%.[26] These data imply that careful attention to mental health problems, including screening for depression, may also be key interventions to improve HRQoL in people with mild to moderate CKD.

Being able to carry out activities of daily living (ADLs) is an important part of living independently. There is a recognised association between having difficulty with ADLs and a lower HRQoL in older adults, though comparison of HRQoL (as assessed by EQ-5D-5L) with FS (as assessed by the KPS) is under-studied.[27] The strong association observed in this study between functional impairment and lower HRQoL including mobility, self care and usual actitivies, even after adjustment for potential confounding factors, suggests that clinicians identifying functional impairment should consider their patient's HRQoL. Previous research has identified higher prevalence of problems with ADLs among people with CKD (defined as eGFR<60ml/min/1.73m^2) than people without, variously reported as between 26% and 55% depending on the nature of the population studied and the measure of ADL used.[28-30] Two large longitudinal studies have shown, in keeping with our findings, a significant reduction in instrumental ADLs and basic ADLs when renal function deteriorates over time.[28,29] It should also be noted that the KPS is a clinician-assessed tool comprising an element of subjectivity and therefore may be less accurate than an in-depth questionnaire looking at both instrumental ADLs and generic ADLs.

In 2016, NICE published their first guideline for the management of individuals with multiple co-existing chronic diseases.[31] These guidelines ask clinicians to consider the overall burdens of disease and treatment, and to discuss these with patients and develop individualised management plans. Amongst other things, they suggest that clinicians assess for frailty (using measures such as gait speed or self-reported health status) and remain particularly vigilant for issues with mental health or chronic pain.[31] Our data provide evidence that a similar approach is warranted in people with mild to moderate CKD. When managing these patients, clinicians should consider comorbidities and discuss how treatments will fit in with those for other comorbidities, particularly mental health and chronic pain-related conditions and those affecting mobility. Obesity, as a potentially modifiable factor with an independent association with both functional impairment and HRQoL in our study, is also an important consideration. This could include devising management plans jointly with other healthcare professionals, streamlining health services to cater for people with multiple conditions, and signposting to appropriate services. Both General Practitioners and nephrologists should be well-placed to manage patients holistically, but patients would benefit from mental health, comorbidity and pain issues being considered in clinical guidelines and outcome

For peer review only - http://bmjopen.bmj.com/site/about/guidelines.xhtml

measures. It is also important to consider the capacity of people to self-manage, and the extent of patient activation, if mental health issues or multiple comorbidities are present.

CONCLUSION

We have observed that people with mild to moderate CKD commonly have multiple comorbidities and many report some HRQoL problems and functional impairment. Taken together with previous observations that the risk of CKD progression is low in this context, these data suggest that mild to moderate CKD in older people could be more important as a marker of increased comorbidity than as a risk factor for ESKD. Clinicians should therefore carefully consider comorbidities and discuss how treatments will fit in with those for other comorbidities, particularly mental health, chronic pain-related conditions and those affecting functional status.

For peer review only

Competing interest statement
None of the authors have any conflicts of interest to declare. The results presented in this paper have not been published previously in whole or part, except in abstract format.

Author contributions
SDSF, JB, PJR, JEM and HMY designed and undertook the analyses. AS and MWT obtained the cohort five year follow up data. MWT, NJM, RJF, and CWM established the RRID cohort, provided data and contributed development of the project and critically reviewed the manuscript. All authors critically reviewed the paper; were involved in the drafting and approval of the final manuscript; and act as guarantors. All authors take responsibility for the data and research governance.

Acknowledgements
The authors acknowledge the contributions of the participants, their families and the participating general practice surgeries without whom this work would not have been possible. The authors would like to thank Shihua Zu who provided advice on using EQ-5D-5L.

Funding
The RRID study was funded by a Research Project Grant (R302/0713) from the Dunhill Medical Trust (http://www.dunhillmedical.org.uk). Previous study funding includes a joint British Renal Society (http://www.britishrenal.org) and Kidney Research UK (http://www.kidneyresearchuk.org) fellowship (BRS3/2008, to NJM), and an unrestricted educational grant (EPWE124712-G) from Roche Products Ltd. (https://www.roche.co.uk). The funders had no role in study design, data collection and analysis, decision to publish, or preparation of the manuscript.

Ethics approval
The RRID study was approved by the Nottingham Research Ethics Committee 1 and was included on the National Institute for Health Research (NIHR) Clinical Research Portfolio (NIHR Study ID: 6632). All participants provided written, informed consent. The RRID study complies with the Declaration of Helsinki and the principles of Good Clinical Practice.

Data sharing statement
Anonymised data can be made available to researchers who meet the conditions of the ethics approval and research governance policy that applies to this study. Researchers may apply for data access by contacting Dr. Teresa Grieve, Research and Development Manager, University Hospitals of Derby and Burton NHS Foundation Trust (teresa.grieve@nhs.net).

Transparency statement
We affirm that the manuscript is an honest, accurate, and transparent account of the study being reported. No important aspects of the study have been omitted and any discrepancies from the study as originally planned have been explained.

For peer review only

For peer review only - http://bmjopen.bmj.com/site/about/guidelines.xhtml

Figure legends

Figure 1. Flow chart of study participants

Figure 2. Relationship between quality of life and functional status

2a. Functional status (by Karnofsky score) and quality of life (by EQ-5D-3L Index score)
2b. Functional status (by Karnofsky score) and quality of life (by EQ-5D self-reported VAS scale)

For peer review only

REFERENCES

1. Hill NR, Fatoba ST, Oke JL, et al. Global Prevalence of Chronic Kidney Disease – A Systematic Review and Meta-Analysis. *PLoS One* 2016; 11(7): e0158765
2. Fraser SDS, Aitken GR, Taal MW, et al. Exploration of chronic kidney disease prevalence estimates using new measures of kidney function in the Health Survey for England. *PLoS One* 2015; 10(2): e0118676
3. NICE Clinical Guidelines 182. Chronic Kidney Disease: Early Identification and Management of Chronic Kidney Disease in Adults in Primary and Secondary Care. London: National Institute of Health and Care Excellence; 2014.
4. Shardlow A, McIntyre NJ, Fluck RJ, McIntyre CW, Taal MW. Chronic Kidney Disease in Primary Care: Outcomes after Five Years in a Prospective Cohort Study. *PLoS Med* 2016;13(9):e1002128
5. Tonelli M, Wiebe N, Guthrie B, et al. Comorbidity as a driver of adverse outcomes in people with chronic kidney disease. *Kidney Int* 2015;88:859-866.
6. Fraser SDS, Roderick PJ, May CR, et al. The burden of comorbidity in people with chronic kidney disease stage 3: a cohort study. *BMC Nephrol* 2015;16:193.
7. Jesky MD, Dutton M, Dasgupta I, et al. Health-Related Quality of Life Impacts Mortality but Not Progression to End-Stage Renal Disease in Pre-Dialysis Chronic Kidney Disease: A Prospective Observational Study. *PLoS One* 2016;11(11):e0165675.
8. Roberti J, Cummings A, Myall M, et al. Work of being an adult patient with chronic kidney disease: a systematic review of qualitative studies. BMJ Open 2018;8(9):e023507.
9. McIntyre N, Fluck R, McIntyre CW, Taal MW. Risk profile in chronic kidney disease stage 3: older versus younger patients. *Nephron Clin Pract* 2011;119(4):269- 276.
10. Herdman M, Gudex C, Lloyd A, Janssen MF, Kind P, Parkin D. Development and preliminary testing of the new five-level version of EQ-5D (EQ-5D-5L). *Qual Life Res* 2011; 20(10):1727-1736.
11. Devlin N, Shah K, Feng Y, Mulhern B, van Hout B. Valuing health-related quality of Life: An EQ-5D-5L Value Set for England. *Health Econ* 2018.27(1):7-22.
12. NICE position statement on use of the EQ-5D-5L value set for England. National Institute of Health and Care Excellence (updated October 2019). https://www.nice.org.uk/about/what-we-do/our-programmes/nice-guidance/technology-appraisal-guidance/eq-5d-5l. Accessed 26th March 2020
13. EuroQol EQ-5D-5L Crosswalk Index Value Calculator. https://euroqol.org/eq-5d-instruments/eq-5d-5l-about/valuation-standard-value-sets/crosswalk-index-value-calculator/. Accessed 26th March 2020
14. van Hout B, Janssen MF, Feng YS, et al. Interim scoring for the EQ-5D-5L: mapping the EQ-5D-5L to EQ-5D-3L value sets. *Value Health*. 2012:15(5):708-15
15. Karnofsky D, Abelmann W, Craver L, Burchenal J. The use of nitrogen mustards in the palliative treatment of cancer. *Cancer* 1948, 1: 634-656.
16. Salloum RG, Smith TJ, Jensen GA, et al. Using Claims-Based Measures to Predict Performance Status Score in Lung Cancer Patients *Cancer* 2011;117(5):1038- 48
17. KDIGO 2012 clinical practice guideline for the evaluation and management of chronic kidney disease. *Kidney Int Suppl*. 2013;3(1):150.
18. Pinto-Sietsma S, Navis G, Janssen W et al. A central body fat distribution is related to renal function impairment, even in lean subjects. *Am J Kidney Dis*. 2003;41:733-741
19. Jordan H, Roderick P, Martin D. The index of multiple deprivation 2000 and accessibility effects on health. *J Epidemiol Community Health*. 2004;58:250-7.

For peer review only - http://bmjopen.bmj.com/site/about/guidelines.xhtml

20. Feng Y, Devlin N, Herdman M. Assessing the health of the general population in England: how do the three- and five-level versions of EQ-5D compare? *Health Qual Life Outcomes* 2015;13:171
21. Breeze E, Jones DA, Wilkinson P et al. Area deprivation, social class, and quality of life among people aged 75 years and over in Britain. *Int J Epidemiol*. 2005;34(2):276-83.
22. de Lusignan SD, Gallagher H, Jones S et al. Audit-based education lowers systolic blood pressure in chronic kidney disease: the Quality Improvement in CKD (QICKD) trial results. *Kidney Int*. 2013;84(3):609-20.
23. Major RW, Brown C, Shepherd D et al. The Primary-Secondary Care Partnership to Improve Outcomes in Chronic Kidney Disease (PSP-CKD) Study: A Cluster Randomized Trial in Primary Care. *J Am Soc Nephrol* 2019;30(7):1261-1270.
24. Bridges S. Mental Health Problems. Health Survey for England 2014. Vol 1 Ch 2. The Health and Social Care Information Centre 2015.
25. Palmer S, Vecchio M, Craig J, et al. Prevalence of depression in chronic kidney disease : systematic review and meta-analysis of observational studies. *Kidney Int* 2013;84(1):179-91.
26. Fischer MJ, Xie D, Jordan N et al. CRIC Study Group Investigators. Factors associated with depressive symptoms and use of antidepressant medications among participants in the Chronic Renal Insufficiency Cohort (CRIC) and Hispanic-CRIC Studies. *Am J Kidney Dis*. 2012 ;60(1):27-38.
27. Lyu W, Wolinsky F. The onset of ADL difficulties and changes in health-related quality of life. *Health Qual Life Outcomes* 2017;15(1):217.
28. Bowling C, Sawyer P, Campbell R, Ahmed A, Allman RM. Impact of chronic kidney disease on activities of daily living in community-dwelling older adults. *J Gerontol A Biol Sci Med Sci* 2011;66(6):689-94.
29. Chin H, Ahn S, Ryu J et al. Renal function and decline in functional capacity in older adults. *Age Ageing* 2014;43:833-8.
30. Smyth A, Glynn L, Murphy A. Mild chronic kidney disease and functional impairment in community-dwelling older adults. *Age Ageing* 2013;42:488-94.
31. National Institute for Health and Care Excellence (NICE). Multimorbidity: clinical assessment and management (NG56). Sept 2016.

For peer review only

For peer review only - http://bmjopen.bmj.com/site/about/guidelines.xhtml

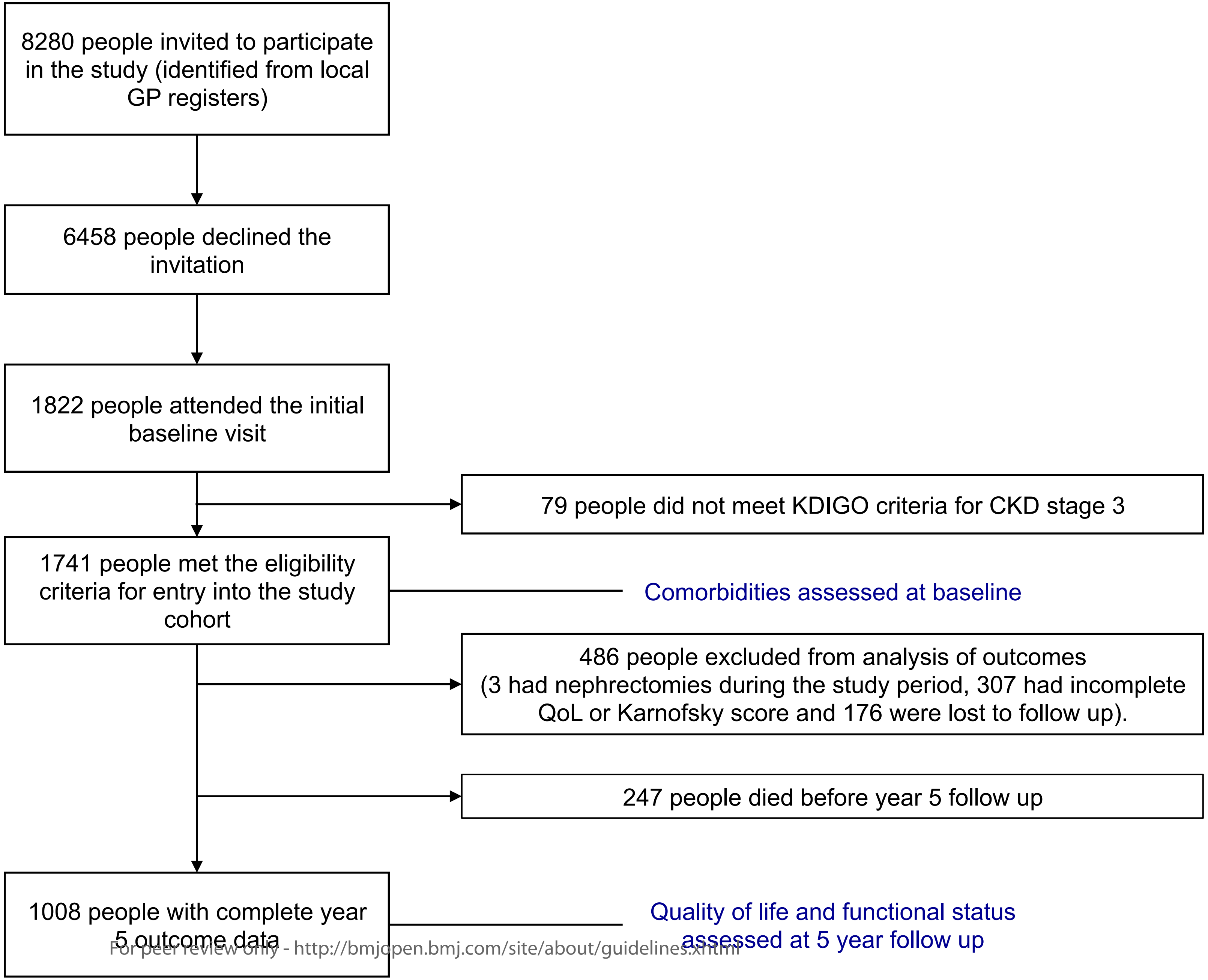

For peer review only - http://bmjopen.bmj.com/site/about/guidelines.xhtml

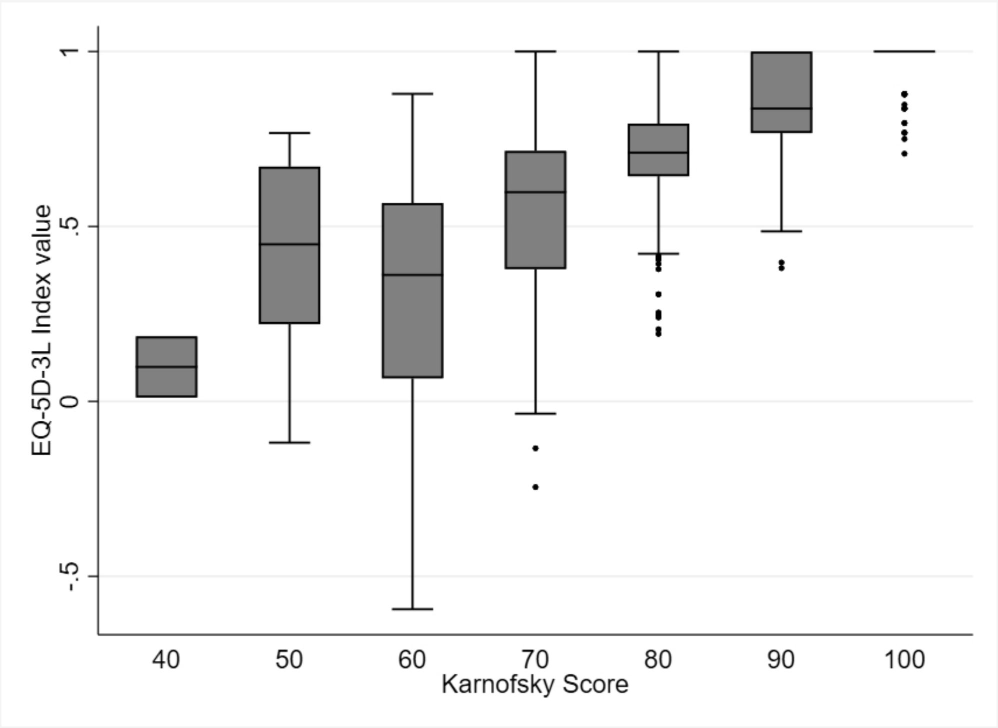

Figure 2. Relationship between quality of life and functional status

2a. Functional status (by Karnofsky score) and quality of life (by EQ-5D-3L Index score)

303x220mm (72 x 72 DPI)

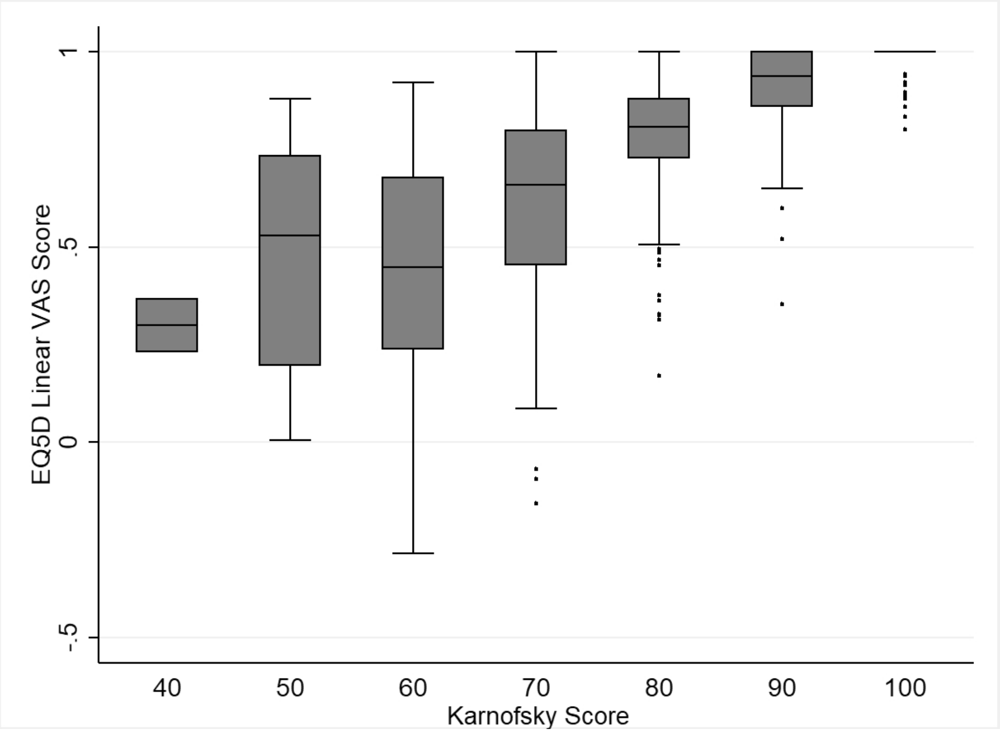

2b. Functional status (by Karnofsky score) and quality of life (by EQ-5D self-reported VAS scale)

390x283mm (72 x 72 DPI)

Supplementary material

Table S1. Characteristics of those with and without complete follow up data at five years.

	Complete HRQoL data (n=1008)	Incomplete HRQoL data (n=486)
**Age (mean(SD))**	70.6 (8.6)	74.7 (9.0)
**Sex (n(%) male)**	387 (38)	160 (33)
**Index of multiple deprivation (IMD) (n (%) in most deprived quintile)**	82 (8)	49 (10)
**Comorbidities (n (%) with three or more)**	344 (34)	206 (42)
**Functional impairment (KPS ≤70)**	234 (23)	2 (0.4%)

Table S2. Associations between patient-reported EQ-5D-5L quality of life domains and clinician-assessed functional status.

Patient-reported EQ-5D-5L quality of life domain		No clinician-assessed functional impairment (KPS score >70)		Clinician-assessed functional impairment (KPS score ≤70)		Total RRID cohort		p**
		n	%*	n	%*	n	%*	
**Mobility**	1 (no problems in walking about)	416	53.8	10	4.3	426	42.3	<0.001
	2 – 5 (some problems)	358	46.2	224	95.7	582	57.7	
**Self care**	1 (no problems washing or dressing)	726	93.8	116	49.6	842	83.5	<0.001
	2 – 5 (some problems)	48	6.2	118	50.4	166	16.5	
**Usual activities**	1 (no problems doing usual activities)	508	65.6	34	14.5	542	53.8	<0.001
	2 – 5 (some problems)	266	34.4	200	85.5	466	46.2	
**Pain/ discomfort**	1 (no pain or discomfort)	270	34.9	26	11.1	296	29.4	<0.001
	2 – 5 (some pain or discomfort)	504	65.1	208	88.9	712	70.6	
**Anxiety/ depression**	1 (not anxious or depressed)	576	74.4	113	48.3	689	68.4	<0.001
	2 – 5 (some anxiety or depression)	198	25.6	121	51.7	319	31.6	
**EQ-5D-3L index score (converted from EQ-5D-5L)**	> median for age and sex	351	45.4	27	11.5	378	37.5	<0.001
	≤ median for age and sex	423	54.7	207	88.5	630	62.5	
**Total**		774	100.0	234	100.0	1008	100.0	

For peer review only - http://bmjopen.bmj.com/site/about/guidelines.xhtml

* Column percentages are shown.** Chi-square test was performed. Abbreviations: KPS= Karnofsky Performance Status, RRID= Renal Risk in Derby

Tables S3 to S6. Logistic regression models examining associations between lower quality of life (EQ-5D-5L domains of usual activities, self-care, pain/discomfort and anxiety/depression categorised as 'no problems' vs. 'any problems') and patient characteristics. N=1008 in univariable models unless otherwise stated, N=1005 for final multivariable logistic regression models

S3. USUAL ACTIVITIES

		Univariable		Multivariable*	
		**OR (95% CI)**	**p**	**OR (95% CI)**	**p**
**Age (years)**		1.03 (1.02 – 1.05)	<0.001	1.02 (1.00 – 1.04)	0.056
**Female sex (vs. male)**		1.05 (0.81 – 1.36)	0.71	-	-
**Index of multiple deprivation (IMD quintile relative to England) (vs. quintile 5: least deprived) [N=1006]**	Quintile 1 (most deprived)	1.45 (0.87 – 2.40)	0.009#	0.83 (0.46 – 1.52)	0.076#
	Quintile 2	1.93 (1.34 – 2.78)		1.64 (1.08 – 2.50)	
	Quintile 3	1.43 (0.97 – 2.12)		1.37 (0.87 – 2.15)	
	Quintile 4	1.25 (0.87 – 1.78)		1.12 (0.74 – 1.69)	
**Number of comorbidities (vs. no comorbidities)**	One	1. 50 (0.77 – 2.91)	<0.001#	1.41 (0.68 – 2.90)	<0.001#
	Two	2.56 (1.32 – 4.97)		1.82 (0.88 – 3.78)	
	Three or more	6.34 (3.28 – 12.25)		4.20 (2.02 – 8.74)	
**Functional status (KPS score) (vs. KPS >70)**	Functional impairment (KPS ≤70)	11.23 (7.59 – 16.64)	<0.001	8.27 (5.43 – 12.58)	<0.001
**eGFR (ml/min/1.73m²) [N=1007]**		0.99 (0.98 – 1.00)	0.013	1.01 (1.00 – 1.02)	0.139
**uACR (KDIGO categories) (vs. category A1, <3mg/mmol) [N=1007]**	A2 (3-29 mg/mmol)	1.24 (0.92 - 1.68)	0.090#	0.92 (0.63 – 1.34)	0.767#
	A3 (≥30mg/mmol)	1.72 (0.97 – 3.09)		1.19 (0.59 – 2.43)	
**Educational attainment (vs. first or higher degree or NVQ 4-5) [N=1007]**	No formal qualifications	1.88 (1.35 – 2.62)	<0.001#	1.22 (0.82 – 1.82)	0.549#
	GCSE, A level or NVQ 1-3	1.30 (0.90 – 1.87)		1.24 (0.82 – 1.89)	
**BMI (vs. <25 kg/m²))**	Overweight (BMI 25-29.99 kg/m²)	1.54 (1.08 – 2.21)	<0.001#	1.38 (0.92 – 2.06)	0.019#
	Obese (BMI ≥30 kg/m²)	2.81 (1.96 – 4.03)		1.82 (1.19 – 2.76)	
**Smoking status (vs. never smoked)**	Current smoker	0.85 (0.46 – 1.60)	0.421#	-	-
	Ex-smoker	1.15 (0.89 – 1.49)		-	

*Adjusted for age, deprivation level, number of comorbidities, functional status, eGFR at five-year follow-up, uACR at five-year follow-up, educational attainment, and BMI. #p value for trend. Abbreviations: OR= Odds Ratio, CI= Confidence Interval, KPS= Karnofsky Performance Status, eGFR= estimated Glomerular Filtration Rate, uACR= Urinary Albumin to Creatinine Ratio, KDIGO= Kidney Disease Improving Global Outcomes,

For peer review only - http://bmjopen.bmj.com/site/about/guidelines.xhtml

For peer review only

GCSE= General Certificate of Secondary Education, A level= Advanced level, NVQ= National Vocational Qualifications, BMI= Body Mass Index, IMD= Index of Multiple Deprivation.

S4. SELF CARE

		Univariable		Multivariable*	
		OR (95% CI)	p	OR (95% CI)	p
**Age (years)**		1.01 (0.99 – 1.03)	0.472	-	-
**Female sex (vs. male)**		0.91 (0.64 – 1.27)	0.568	-	-
**Index of multiple deprivation (IMD quintile relative to England) (vs. quintile 5: least deprived) [N=1006]**	Quintile 1 (most deprived)	2.27 (1.19 – 4.34)	0.041#	1.12 (0.51 – 2.42)	0.895#
	Quintile 2	1.90 (1.15 – 3.15)		1.20 (0.67 – 2.19)	
	Quintile 3	1.64 (0.95 – 2.84)		1.31 (0.68 – 2.51)	
	Quintile 4	1.26 (0.75 – 2.13)		0.99 (0.54 – 1.85)	
**Number of comorbidities (vs. no comorbidities)**	One	1.43 (0.41 – 4.92)	<0.001#	1.27 (0.34 – 4.84)	0.050#
	Two	3.37 (1.01 – 11.21)		1.89 (0.51 – 6.97)	
	Three or more	6.45 (1.97 – 21.15)		2.64 (0.72 – 9.67)	
**Functional status (KPS score) (vs. KPS >70)**	Functional impairment (KPS ≤70)	15.39 (10.43 – 22.69)	<0.001	13.08 (8.46 – 20.22)	<0.001
**eGFR (ml/min/1.73m^2) [N=1007]**		0.99 (0.98 – 1.00)	0.055	1.01 (0.99 – 1.21)	0.154
**uACR (KDIGO categories) (vs. category A1, <3mg/mmol) [N=1007]**	A2 (3-29 mg/mmol)	1.37 (0.93 - 2.03)	0.223 #	-	-
	A3 (≥30mg/mmol)	1.39 (0.67 – 2.86)		-	
**Educational attainment (vs. first or higher degree or NVQ 4-5) [N=1007]**	No formal qualifications	1.91 (1.18 – 3.08)	0.014#	1.11 (0.63 – 1.97)	0.669#
	GCSE, A level or NVQ 1-3	1.31 (0.76 – 2.23)		1.32 (0.69 – 2.50)	
**BMI (vs. <25 kg/m^2))**	Overweight (BMI 25-29.99 kg/m^2)	1.20 (0.68 – 2.13)	<0.001#	0.95 (0.49 – 1.82)	0.003#
	Obese (BMI ≥30 kg/m^2)	3.47 (2.03 – 5.92)		1.98 (1.06 – 3.71)	
**Smoking status (vs. never smoked)**	Current smoker	2.17 (1.05 – 4.51)	0.018#	2.53 (1.00 – 6.40)	0.006#
	Ex-smoker	1.53 (1.08 – 2.16)		1.89 (1.24 – 2.88)	

* Adjusted for deprivation level, number of comorbidities, functional status, eGFR at five-year follow-up, educational attainment, BMI and smoking. #p value for trend. Abbreviations: OR= Odds Ratio, CI= Confidence Interval, KPS= Karnofsky Performance Status, eGFR= estimated Glomerular Filtration Rate, uACR= Urinary Albumin to Creatinine Ratio, KDIGO= Kidney Disease Improving Global Outcomes, GCSE= General Certificate of Secondary Education, A level= Advanced level, NVQ= National Vocational Qualifications, BMI= Body Mass Index, IMD= Index of Multiple Deprivation.

For peer review only

S5. PAIN / DISCOMFORT

		Univariable		Multivariable*	
		OR (95% CI)	p	OR (95% CI)	p
**Age (years)**		1.02 (1.00 – 1.03)	0.026	1.01 (0.99 – 1.02)	0.550
**Female sex (vs. male)**		1.23 (0.93 – 1.63)	0.141	-	-
**Index of multiple deprivation (IMD quintile relative to England) (vs. quintile 5: least deprived) [N=1006]**	Quintile 1 (most deprived)	2.42 (1.30 – 4.50)		1.87 (0.97 – 3.60)	
	Quintile 2	1.52 (1.03 – 2.24)	0.046#	1.33 (0.88 – 2.03)	0.381#
	Quintile 3	1.27 (0.84 – 1.92)		1.26 (0.81 – 1.95)	
	Quintile 4	1.25 (0.86 – 1.82)		1.24 (0.83 – 1.84)	
**Number of comorbidities (vs. no comorbidities)**	One	1.62 (0.91 – 2.86)		1.49 (0.82 – 2.71)	
	Two	2.95 (1.65 – 5.29)	<0.001#	2.27 (1.22 – 4.20)	<0.001#
	Three or more	4.70 (2.60 – 8.48)		3.06 (1.63 – 5.73)	
**Functional status (KPS score) (vs. KPS >70)**	Functional impairment (KPS ≤70)	4.29 (2.78 – 6.61)	<0.001	2.94 (1.86 – 4.67)	<0.001
**eGFR (ml/min/1.73m²) [N=1007]**		0.99 (0.98 – 1.00)	0.150	-	-
**uACR (KDIGO categories) (vs. category A1, <3mg/mmol) [N=1007]**	A2 (3-29 mg/mmol)	0.87 (0.62 - 1.20)	0.670#	-	-
	A3 (≥30mg/mmol)	1.04 (0.55 – 1.96)		-	
**Educational attainment (vs. first or higher degree or NVQ 4-5) [N=1007]**	No formal qualifications	1.67 (1.19 – 2.35)	0.007#	1.23 (0.84 – 1.97)	0.075#
	GCSE, A level or NVQ 1-3	1.65 (1.13 – 2.41)		1.59 (1.06 – 2.38)	
**BMI (vs. <25 kg/m²))**	Overweight (BMI 25-29.99 kg/m²)	1.63 (1.15 – 2.30)	<0.001#	1.47 (1.02 – 2.12)	<0.001#
	Obese (BMI ≥30 kg/m²)	3.26 (2.23 – 4.76)		2.37 (1.58 – 3.55)	
**Smoking status (vs. never smoked)**	Current smoker	1.12 (0.56 – 2.24)	0.945#	-	-
	Ex-smoker	1.02 (0.77 – 1.34)		-	

Adjusted for age, deprivation level, number of comorbidities, functional status, educational attainment, and BMI. #p value for trend. Abbreviations: OR= Odds Ratio, CI= Confidence Interval, KPS= Karnofsky Performance Status, eGFR= estimated Glomerular Filtration Rate, uACR= Urinary Albumin to Creatinine Ratio, KDIGO= Kidney Disease Improving Global Outcomes, GCSE= General Certificate of Secondary Education, A level= Advanced level, NVQ= National Vocational Qualifications, BMI= Body Mass Index, IMD= Index of Multiple Deprivation.

For peer review only

For peer review only - http://bmjopen.bmj.com/site/about/guidelines.xhtml

S6. ANXIETY / DEPRESSION

		Univariable		Multivariable*	
		OR (95% CI)	p	OR (95% CI)	p
**Age (years)**		1.00 (0.98 – 1.01)	0.942	-	-
**Female sex (vs. male)**		1.63 (1.23 – 2.16)	0.001	1.60 (1.18 –2.16)	0.002
**Index of multiple deprivation (IMD quintile relative to England) (vs. quintile 5: least deprived) [N=1006]**	Quintile 1 (most deprived)	0.89 (0.51 – 1.54)	0.856#	-	-
	Quintile 2	0.94 (0.64 – 1.38)		-	
	Quintile 3	1.14 (0.76 – 1.72)		-	
	Quintile 4	0.95 (0.65 – 1.38)		-	
**Number of comorbidities (vs. no comorbidities)**	One	0.80 (0.43 – 1.51)	0.005#	0.94 (0.49 – 1.81)	0.269#
	Two	1.25 (0.67 – 2.34)		1.29 (0.67 – 2.47)	
	Three or more	1.48 (0.80 – 2.75)		1.30 (0.68 – 2.49)	
**Functional status (KPS score) (vs. KPS >70)**	Functional impairment (KPS ≤70)	3.12 (2.30 – 4.22)	<0.001	3.08 (2.23 – 4.27)	<0.001
**eGFR (ml/min/1.73m²) [N=1007]**		1.00 (0.99 – 1.01)	0.998	-	-
**uACR (KDIGO categories) (vs. category A1, <3mg/mmol) [N=1007]**	A2 (3-29 mg/mmol)	1.00 (0.72 - 1.38)	0.851#	-	-
	A3 (≥30mg/mmol)	0.83 (0.44 – 1.57)		-	
**Educational attainment (vs. first or higher degree or NVQ 4-5) [N=1007]**	No formal qualifications	1.51 (1.04 – 2.18)	0.009#	1.05 (0.71 – 1.56)	0.009#
	GCSE, A level or NVQ 1-3	1.86 (1.25 – 2.77)		1.67 (1.10 – 2.52)	
**BMI (vs. <25 kg/m²))**	Overweight (BMI 25-29.99 kg/m²)	0.94 (0.65 – 1.36)	0.133#	-	-
	Obese (BMI ≥30 kg/m²)	1.26 (0.87 – 1.82)		-	
**Smoking status (vs. never smoked)**	Current smoker	1.00 (0.51 – 1.94)	0.989#	-	-
	Ex-smoker	0.98 (0.75 – 1.29)		-	

* Adjusted for sex, number of comorbidities, and functional status. #p value for trend. Abbreviations: OR= Odds Ratio, CI= Confidence Interval, KPS= Karnofsky Performance Status, eGFR= estimated Glomerular Filtration Rate, uACR= Urinary Albumin to Creatinine Ratio, KDIGO= Kidney Disease Improving Global Outcomes, GCSE= General Certificate of Secondary Education, A level= Advanced level, NVQ= National Vocational Qualifications, BMI= Body Mass Index, IMD= Index of Multiple Deprivation.

For peer review only

For peer review only - http://bmjopen.bmj.com/site/about/guidelines.xhtml

STROBE 2007 (v4) checklist of items to be included in reports of observational studies in epidemiology*
Checklist for cohort, case-control, and cross-sectional studies (combined)

Section/Topic	Item #	Recommendation	Reported on page #
**Title and abstract**	1	(*a*) Indicate the study's design with a commonly used term in the title or the abstract	1
		(*b*) Provide in the abstract an informative and balanced summary of what was done and what was found	2
**Introduction**			
Background/rationale	2	Explain the scientific background and rationale for the investigation being reported	3
Objectives	3	State specific objectives, including any pre-specified hypotheses	3
**Methods**			
Study design	4	Present key elements of study design early in the paper	3-5
Setting	5	Describe the setting, locations, and relevant dates, including periods of recruitment, exposure, follow-up, and data collection	3-4
Participants	6	(*a*) *Cohort study*—Give the eligibility criteria, and the sources and methods of selection of participants. Describe methods of follow-up<br>*Case-control study*—Give the eligibility criteria, and the sources and methods of case ascertainment and control selection. Give the rationale for the choice of cases and controls<br>*Cross-sectional study*—Give the eligibility criteria, and the sources and methods of selection of participants	3-4
		(*b*) *Cohort study*—For matched studies, give matching criteria and number of exposed and unexposed<br>*Case-control study*—For matched studies, give matching criteria and the number of controls per case	
Variables	7	Clearly define all outcomes, exposures, predictors, potential confounders, and effect modifiers. Give diagnostic criteria, if applicable	3-5
Data sources/ measurement	8*	For each variable of interest, give sources of data and details of methods of assessment (measurement). Describe comparability of assessment methods if there is more than one group	3-5
Bias	9	Describe any efforts to address potential sources of bias	5
Study size	10	Explain how the study size was arrived at	3
Quantitative variables	11	Explain how quantitative variables were handled in the analyses. If applicable, describe which groupings were chosen and why	3-5
Statistical methods	12	(*a*) Describe all statistical methods, including those used to control for confounding	5
		(*b*) Describe any methods used to examine subgroups and interactions	5
		(*c*) Explain how missing data were addressed	5
		(*d*) *Cohort study*—If applicable, explain how loss to follow-up was addressed<br>*Case-control study*—If applicable, explain how matching of cases and controls was addressed	5

For peer review only

		Cross-sectional study—If applicable, describe analytical methods taking account of sampling strategy	
		(*e*) Describe any sensitivity analyses	5
**Results**			
Participants	13*	(a) Report numbers of individuals at each stage of study—eg numbers potentially eligible, examined for eligibility, confirmed eligible, included in the study, completing follow-up, and analysed	6 and Fig 1
		(b) Give reasons for non-participation at each stage	Fig 1
		(c) Consider use of a flow diagram	Fig 1
Descriptive data	14*	(a) Give characteristics of study participants (eg demographic, clinical, social) and information on exposures and potential confounders	6, Table 1
		(b) Indicate number of participants with missing data for each variable of interest	Complete data only
		(c) *Cohort study*—Summarise follow-up time (eg, average and total amount)	
Outcome data	15*	*Cohort study*—Report numbers of outcome events or summary measures over time	
		Case-control study—Report numbers in each exposure category, or summary measures of exposure	
		Cross-sectional study—Report numbers of outcome events or summary measures	7, Table 1
Main results	16	(*a*) Give unadjusted estimates and, if applicable, confounder-adjusted estimates and their precision (eg, 95% confidence interval). Make clear which confounders were adjusted for and why they were included	8, Table 3
		(*b*) Report category boundaries when continuous variables were categorized	6, Table 1
		(*c*) If relevant, consider translating estimates of relative risk into absolute risk for a meaningful time period	n/a
Other analyses	17	Report other analyses done—eg analyses of subgroups and interactions, and sensitivity analyses	7,8
**Discussion**			
Key results	18	Summarise key results with reference to study objectives	6-11
Limitations	19	Discuss limitations of the study, taking into account sources of potential bias or imprecision. Discuss both direction and magnitude of any potential bias	13
Interpretation	20	Give a cautious overall interpretation of results considering objectives, limitations, multiplicity of analyses, results from similar studies, and other relevant evidence	12-14
Generalisability	21	Discuss the generalisability (external validity) of the study results	13
**Other information**			
Funding	22	Give the source of funding and the role of the funders for the present study and, if applicable, for the original study on which the present article is based	15

*Give information separately for cases and controls in case-control studies and, if applicable, for exposed and unexposed groups in cohort and cross-sectional studies.

Note: An Explanation and Elaboration article discusses each checklist item and gives methodological background and published examples of transparent reporting. The STROBE checklist is best used in conjunction with this article (freely available on the Web sites of PLoS Medicine at http://www.plosmedicine.org/, Annals of Internal Medicine at http://www.annals.org/, and Epidemiology at http://www.epidem.com/). Information on the STROBE Initiative is available at www.strobe-statement.org.

For peer review only

BMJ Open

Health related quality of life, functional impairment and comorbidity in people with mild to moderate chronic kidney disease: a cross sectional study

Journal:	*BMJ Open*
Manuscript ID	bmjopen-2020-040286.R1
Article Type:	Original research
Date Submitted by the Author:	27-Jun-2020

Complete List of Authors:	Fraser, Simon; University of Southampton, Faculty of Medicine<br>Barker, Jenny; University of Southampton, Faculty of Medicine<br>Roderick, Paul; University of Southampton, Faculty of Medicine<br>Yuen, Ho Ming; University of Southampton, Faculty of Medicine<br>Shardlow, Adam; Royal Derby Hospital, Renal Medicine<br>Morris, James; University of Southampton, Faculty of Medicine<br>McIntyre, Natasha; Royal Derby Hospital, Renal Medicine<br>Fluck, Richard; Royal Derby Hospital, Renal Medicine<br>McIntyre, CW; University of Western Ontario, Division of Nephrology<br>Taal, Maarten; Royal Derby Hospital, Renal Medicine; University of Nottingham, Centre for Kidney Research and Innovation

**Primary Subject Heading**:	Renal medicine
Secondary Subject Heading:	Epidemiology, Renal medicine, Public health
Keywords:	Chronic renal failure < NEPHROLOGY, EPIDEMIOLOGY, PRIMARY CARE, PUBLIC HEALTH

SCHOLARONE™
Manuscripts

I, the Submitting Author has the right to grant and does grant on behalf of all authors of the Work (as defined in the below author licence), an exclusive licence and/or a non-exclusive licence for contributions from authors who are: i) UK Crown employees; ii) where BMJ has agreed a CC-BY licence shall apply, and/or iii) in accordance with the terms applicable for US Federal Government officers or employees acting as part of their official duties; on a worldwide, perpetual, irrevocable, royalty-free basis to BMJ Publishing Group Ltd ("BMJ") its licensees and where the relevant Journal is co-owned by BMJ to the co-owners of the Journal, to publish the Work in this journal and any other BMJ products and to exploit all rights, as set out in our licence.

The Submitting Author accepts and understands that any supply made under these terms is made by BMJ to the Submitting Author unless you are acting as an employee on behalf of your employer or a postgraduate student of an affiliated institution which is paying any applicable article publishing charge ("APC") for Open Access articles. Where the Submitting Author wishes to make the Work available on an Open Access basis (and intends to pay the relevant APC), the terms of reuse of such Open Access shall be governed by a Creative Commons licence – details of these licences and which Creative Commons licence will apply to this Work are set out in our licence referred to above.

Other than as permitted in any relevant BMJ Author's Self Archiving Policies, I confirm this Work has not been accepted for publication elsewhere, is not being considered for publication elsewhere and does not duplicate material already published. I confirm all authors consent to publication of this Work and authorise the granting of this licence.

For peer review only

Health related quality of life, functional impairment and comorbidity in people with mild to moderate chronic kidney disease: a cross sectional study

Corresponding author:

Simon DS Fraser[1], School of Primary Care, Population Sciences and Medical Education, Faculty of Medicine, University of Southampton, South Academic Block, Southampton General Hospital, Tremona Road, Southampton, Hampshire, UK, SO16 6YD. email: s.fraser@soton.ac.uk

Co-authors:

Jenny Barker[1]
Paul J Roderick[1]
Ho Ming Yuen[1]
Adam Shardlow[2]
James E Morris[1]
Natasha J McIntyre[2]
Richard J Fluck[3]
Chris W McIntyre[4]
Maarten W Taal[2]

Institutions:

1. School of Primary Care, Population Sciences and Medical Education, Faculty of Medicine, University of Southampton, South Academic Block, Southampton General Hospital, Tremona Road, Southampton, Hampshire, UK, SO16 6YD.
2. Division of Medical Sciences and Graduate-Entry Medicine, University of Nottingham, Derby, UK
3. The Department of Renal Medicine, Royal Derby Hospital NHS Foundation Trust, Derby, Derbyshire, UK
4. Department of Medical Biophysics, Medical Sciences Building, Western University, London, Ontario, Canada

Word count (excluding title page, abstract, references, figures and tables): **3666**

For peer review only - http://bmjopen.bmj.com/site/about/guidelines.xhtml

Abstract

Objectives
To determine the associations between comorbidities, health related quality of life (HRQoL) and functional impairment in people with mild to moderate chronic kidney disease (CKD) in primary care.

Design
Cross-sectional analysis at five-year follow-up in a prospective cohort study

Setting
Thirty- two general practitioner (GP) surgeries in England

Participants
1008 participants with CKD stage 3 (of 1741people recruited at baseline in the Renal Risk in Derby study) who survived to five years and had complete follow-up data for HRQoL and functional status (FS).

Primary and secondary outcome measures
HRQoL assessed using the EQ-5D-5L (using EQ-5D-5L domains of mobility, self care, usual activities, pain/discomfort and anxiety/depression and index value using utility scores calculated from the English general population), and FS using the Karnofsky Performance Status scale (functional impairment defined as Karnofksy score ≤70). Comorbidity was defined by self-reported or doctor-diagnosed condition, disease-specific medication or blood result.

Results
Mean age was 75.8 years. The numbers reporting some problems in EQ-5D-5L domains were: 582 (57.7%) for mobility, 166 (16.5%) for self-care, 466 (46.2%) for usual activities, 712 (70.6%) for pain/discomfort, and 319 (31.6%) for anxiety/depression. Only 191 (18.9%) reported no problems in any domain. HRQoL index values showed greater variation among those with lower FS (for example, for those with Karnofsky score of 60, the median (interquartile range) EQ-5D index value was 0.45 (0.24 to 0.68) compared with 0.94 (0.86 to 1) for those with Karnofsky score of 90). Overall, 234 (23.2%) had functional impairment.
In multivariable logistic regression models functional impairment was independently associated with experiencing problems for all EQ-5D-5L domains (mobility: odds ratio (OR) 16.87 (95% confidence interval (CI) 8.70 to 32.79, p<0.001, self care: OR 13.08 (95% CI 8.46 to 20.22), p<0.001, usual activities: OR 8.27 (95% CI 5.43 to 12.58), p<0.001, pain/discomfort: OR 2.94 (95%CI 1.86 to 4.67), p<0.001, anxiety/depression: 3.08 (95%CI 2.23 to 4.27), p<0.001). Higher comorbidity count and obesity were independently associated with problems in mobility, self-care, usual activities and pain/discomfort: for three or more comorbidities vs. none: (mobility: OR 2.10 (95%CI 1.08 to 4.10, p for trend 0.002), self care: OR 2.64 (95%CI 0.72 to 9.67, p for trend 0.05), usual activities: OR 4.20 (95% CI 2.02 to 8.74, p for trend <0.001), pain/discomfort: OR 3.06 (95%CI 1.63 to 5.73, p for trend <0.001, and for obese (body mass index (BMI) ≥30 kg/m^2) vs. BMI <25 kg/m^2: (mobility: OR 2.44 (95%CI 1.61 to 3.69, p for trend <0.001), self care: OR 1.98 (95%CI 1.06 to 3.71, p for trend 0.003), usual activities: OR 1.82 (95% CI 1.19 to 2.76, p for trend 0.019), pain/discomfort: OR 2.37 (95%CI 1.58 to 3.55, p for trend <0.001. Female sex, lower FS and lower educational attainment were independently associated with anxiety/depression (ORs 1.60 (95% CI 1.18 to 2.16, p 0.002), 3.08 (95% CI 2.23 to 4.27, p <0.001), and 1.67 (95%CI 1.10 to 2.52, p 0.009) respectively). Older age, higher comorbidity count, albuminuria (≥30mg/mmol vs. <3mg/mmol), lower educational attainment (no formal qualifications vs. degree level) and obesity were independently associated with

For peer review only - http://bmjopen.bmj.com/site/about/guidelines.xhtml

functional impairment (ORs 1.07 (95% CI 1.04 to 1.09, p <0.001), 2.18 (95%CI 0.80 to 5.96, p for trend <0.001), 1.74 (95%CI 0.82 – 3.68, p for trend 0.005), 2.08 (95%CI 1.26 to 3.41, p for trend <0.001), and 4.23 (95%CI 2.48 to 7.20) respectively).

Conclusions

The majority of persons with mild to moderate CKD reported reductions in at least one HRQoL domain, which were independently associated with comorbidities, obesity and functional impairment.

Trial registration number

National Institute for Health Research Clinical Research Portfolio Study Number 6632

For peer review only

For peer review only - http://bmjopen.bmj.com/site/about/guidelines.xhtml

Strengths and limitations of this study

- This study involved a large cohort of people with CKD recruited from primary care, a setting in which patients with mild to moderate CKD are typically managed in the UK.
- A broad range of comorbidities were included but they were identified at baseline only, not at follow up, by which time the number of comorbidities may have changed.
- Health related quality of life and functional status were measured in the same patient group and the use of the EQ-5D-5L index measure and data from the HSE enabled comparison with a general population.
- Health related quality of life and functional status measures were taken at five-year follow-up but not at baseline and we were therefore unable to identify change over time.
- This was a cross-sectional study of survivors and we are therefore not able to draw causative links.

For peer review only

For peer review only - http://bmjopen.bmj.com/site/about/guidelines.xhtml

INTRODUCTION

Chronic kidney disease (CKD) is common globally, affecting about 13% of the general adult population, with CKD stage 3 the most prevalent category.[1,2] Current treatment guidelines for CKD are disease-specific and focus on reducing progression and preventing complications such as cardiovascular disease.[3] However, in the UK most people with CKD stage 3 are managed in primary care and in this context only a minority (18%) evidence progression over 5 years.[4] The risk of end-stage kidney disease (ESKD) is extremely low (0.2%).[4]

Conversely, comorbidities (additional chronic diseases) are common in individuals with CKD and can worsen clinical outcomes and health related quality of life (HRQoL).[5] 96% of people with stage 3 disease have at least one comorbidity, around 40% have a comorbidity count of two or more.[6]

A significant body of research has explored HRQoL and the functional status (FS) of people with ESKD or following kidney transplant but these factors are not well explored in those with less severe CKD. Among 733 people with high risk CKD in the Renal Impairment In Secondary Care (RIISC) study, 555 (76%) reported problems in one or more of the EQ5D domains.[7,8]

This is a clinically important knowledge gap because mild to moderate reductions in glomerular filtration rate (GFR) are usually asymptomatic, so improved understanding of the comorbidities and symptoms that affect HRQoL and FS in this group of people is important to facilitate a holistic approach to management. The objective of this study was therefore to evaluate HRQoL and determine the associations between comorbidities, HRQoL and functional impairment in people with mild to moderate CKD in primary care.

MATERIALS AND METHODS

A detailed description of the Renal Risk in Derby (RRID) study methodology has been published elsewhere.[9] In summary, approximately 8,280 people with CKD stage 3 were identified from renal registers at 32 primary care clinics in Derbyshire, UK between 2008 and 2010 and invited to participate in the study. Of these people, 1,822 attended initial baseline visits and 1,741 met eligibility criteria (age ≥ 18 years; two estimated GFR (eGFR) results (derived from the Modification of Diet in Renal Disease study [MDRD] equation) of 30–59 ml/min/1.73 m^2, at least 90 days apart).[9] People with a life expectancy of less than one year, who were unable to attend study visits, or who had a solid organ transplant were excluded.

Health related quality of life

HRQoL was assessed at five-year follow-up using the EQ-5D-5L, a widely used, validated measure of health status that can be standardised to different populations. EQ-5D-5L consists of two aspects: a descriptive system, in which participants are asked to rate their health state from 1-5 against five domains (mobility, self-care, usual activities, pain/discomfort, anxiety/depression) and the EQ visual analogue scale (EQ-VAS), in which participants rate their health on a scale ranging from 'the best health you can imagine,' (100) to 'the worst health you can imagine,' (0).[10] An EQ-5D-5L value set has previously been published for England.[11] However, concerns have been raised about the quality and reliability of the data collected in the valuation study such that the English National Institute for Health and Care Excellence (NICE) recommend 'If data were gathered using the EQ-5D-5L descriptive system, utility values in reference-case analyses should be calculated by mapping the 5L descriptive system data onto the 3L value set'. [12] For these analyses, individual health states were therefore converted using the Euroqol EQ-5D-5L Crosswalk Index Value Calculator into a single 3L index value (a preference-based score that typically ranges from states worse than dead (<0) to 1 (full health) with dead at 0) using utility scores calculated from the English general

For peer review only - http://bmjopen.bmj.com/site/about/guidelines.xhtml

population.[13,14] The index value and the EQ-VAS score were used to graphically display the relationship between HRQoL and FS.

Functional status

FS, defined in this paper as the physical ability to perform normal activities and independently self-care, was assessed at five-year follow-up using the Karnofsky Performance Status (KPS) scale. The KPS is a clinician-assessed score originally developed in oncology and was used for assessing prognosis and management in cancer patients.[15] The scale ranges from 'normal/ no complaints,' (100) to 'dead' (0). Theoretically the scale can take any whole number value within the range, but in practice results are commonly recorded as multiples of ten, therefore KPS was treated as an ordinal variable in this study. The original continuous KPS score >70 is defined as being 'able to carry on normal activity and to work with no special care needed,' a score of between 50 and 70 inclusive is defined as 'unable to work, able to live at home and care for most personal needs; varying amount of assistance needed', and a score of less than or equal to 40 is defined as 'unable to care for self, requiring the equivalent of institutional or hospital care'.[15] Functional impairment was analysed as a binary outcome due to the small number of patients with low KPS score. A KPS score of ≤ 70 vs. > 70 was chosen to compare those able to carry on normal life with those experiencing some functional impairment as has been used in evaluation of FS in patients with lung cancer.[16]

Comorbidities identified at baseline

The methods for defining comorbidities in participants have been described in detail elsewhere.[6] In brief, eleven comorbidities were pragmatically identified at baseline using information from a combination of sources and agreed by consensus between three clinicians (SF, MWT and PJR): patient questionnaires in which patients were asked to list chronic medications (followed by verbal confirmation with verification of repeat prescriptions where possible), blood pressure measurement at the time of baseline study visit, and self-reported clinical diagnoses. Self-reported comorbidities included heart failure, ischaemic heart disease, peripheral vascular disease (defined as peripheral arterial revascularization or amputation) and cerebrovascular disease (stroke or transient ischaemic attack). Diagnoses of chronic respiratory disorder, depression, painful condition, hypertension, diabetes and thyroid disorders were made according to medication history or patient report. Anaemia was defined according to Kidney Disease Improving Global Outcomes (KDIGO) guidelines as haemoglobin <13.0 g/dl (<130 g/l) in males and <12.0 g/dl (<120 g/l) in females, at baseline.[17] Hypertension was defined either by medication history, or by a systolic blood pressure >140mmHg or diastolic >90mmHg, at baseline.

Kidney function

Kidney function was assessed at five-year follow-up. eGFR was calculated using the Chronic Kidney Disease Epidemiology Collaboration (CKDEPI) equation and was treated as a continuous variable. The urine albumin-to-creatinine ratio (uACR) from three consecutive early morning specimens was used for analysis. uACR was categorised into three levels according to KDIGO guidelines and fitted as a discrete variable in regression analyses.

Methods for defining CKD progression have also been detailed elsewhere.[4] In summary, progression of CKD was defined as a 25% decline in GFR, coupled with a worsening of GFR category or an increase in albuminuria category. CKD remission was defined as the presence of both eGFR >60 ml/min/1.73 m^2 and uACR <3 mg/mmol at any study visit in an individual who had previously met KDIGO diagnostic criteria for CKD.

Other baseline measures

Body mass index (BMI) was calculated from weight in kilograms divided by square of height in metres and was treated as a categorical variable.[18] Smoking status was categorized as never

For peer review only - http://bmjopen.bmj.com/site/about/guidelines.xhtml

smoked, ex-smoker, and current smoker. Socioeconomic status was assessed using self-reported educational attainment (categorized into no formal qualifications, school or equivalent qualifications, and degree or equivalent qualifications) as well as the Index of Multiple Deprivation (IMD) score, categorized in quintiles.[19] The IMD is a measure of relative deprivation for small areas of residence in England and combines information from seven domains: income; employment; education, skills and training; health and disability; crime; barriers to housing and services; and living environment. Self-reported ethnicity status was also collected.

Statistical analyses
Descriptive statistics were used to show the characteristics of the study participants at five-year follow-up. Descriptive statistics were also used to show the distribution of functional impairment (KPS $\leq$70) among those reporting problems in the five EQ-5D-5L domains. Associations between the patient-reported EQ-5D-5L domains and FS was assessed using the Chi-square test. Ratings from the five participant-reported EQ-5D-5L domains were also compared between the RRID cohort and those reported by people aged 65 years and over in the 2012 Health Survey for England (HSE) – which is representative of the England population.[20] A comparison of basic characteristics was also made between those with and without complete five year follow up data.
Univariable logistic regression models were used to assess the relationships between having ‘some problems’ in each EQ-5D-5L domain and each predictor variable, including comorbidity count and year-five eGFR. Variables considered to be clinically relevant and where $p<0.1$ on univariable analysis were subsequently included in multivariable logistic regression models. This process was then repeated for the relationship with the outcome variable functional impairment. Due to the small number of non-white participants, ethnicity was not included in the models.
In the regression models, interactions between the individual and area measures of socioeconomic status were also tested because of the potential for the relationship between individual socioeconomic status (indicated by educational attainment) and HRQoL to vary by area deprivation, particularly for older people.[21] The level of significance was set at 5%. All analyses were performed using Stata/IC version 15.0.
The RRID study was approved by the Nottingham Research Ethics Committee 1 and was included on the National Institute for Health Research (NIHR) Clinical Research Portfolio (NIHR Study ID: 6632). All participants provided written, informed consent. The RRID study complies with the Declaration of Helsinki and the principles of Good Clinical Practice.

Patient and public involvement
The RRID study design was discussed with a patient and two feedback meetings for participants and their families were organised after the year five visits, which were well attended. In addition, a web page provides updates and information for participants: https://www.uhdb.nhs.uk/renal-risk-in-derby-rrid-study/

RESULTS

Of 1741 participants recruited, 1494 survived to five years, and of these 1008 participants (67% of survivors) had complete five-year follow-up data for HRQoL and FS (Figure 1). The mean age of the cohort was 75.8 years (SD 8.6) and the majority (n=621, 61.6%) were female (Table 1).

Table 1. Characteristics of patients at five-year follow-up in the Renal Risk in Derby study, N=1008 unless otherwise stated

**Variable**	**Category**	**Descriptive statistics**
**Age in years**[#], mean (SD)		75.8 (8.6)

For peer review only - http://bmjopen.bmj.com/site/about/guidelines.xhtml

**Age group**[#], n (%)	<70 years	220 (21.8)
	70-80 years	467 (46.3)
	>80 years	321 (31.8)
**Sex***, n (%)	Male	387 (38.4)
	Female	621 (61.6)
**Ethnicity***, n (%)	White	994 (98.6)
	Other**	14 (1.4)
**Educational attainment***, n (%) [N=1007]	No formal qualifications	506 (50.3)
	GCSE, A level, NVQ 1-3	291 (28.9)
	First or higher degree, NVQ 4-5	210 (20.9)
**Index of multiple deprivation** (IMD quintile relative to England)*, n (%) [N=1006]	Quintile 1 (most deprived)	82 (8.2)
	Quintile 2	243 (24.2)
	Quintile 3	184 (18.3)
	Quintile 4	258 (25.6)
	Quintile 5 (least deprived)	239 (23.8)
**Body mass index***, n (%)	Normal or underweight (<25 kg/m^2)	195 (19.3)
	Overweight (25-29.99 kg/m^2)	422 (41.9)
	Obese (≥30 kg/m^2)	391 (38.8)
**Smoking status***, n (%)	Never smoked	496 (49.2)
	Ex-smoker	468 (46.4)
	Current smoker	44 (4.4)
**eGFR**[#] **in mL/min/1.73m^2**, mean (SD) [N=1007]		54.0 (15.2)
**uACR**[#] **in mg/mmol,** median (IQR) [N=1007]		0.7 (0-3.3)
**KDIGO uACR categories**[#] n (%)	A1	741 (73.5)
	A2	217(21.5)
	A3	50 (5.0)
**KDIGO eGFR categories**[#] (eGFR in mL/min/1.73m^2)	G1 (eGFR ≥90)	13 (1.3)
	G2 (eGFR 60-89)	337 (33.4)
	G3a (eGFR 45-59)	379 (37.6)
	G3b (eGFR 30-44)	223 (22.1)
	G4 (eGFR 15-29)	55 (5.5)
	G5 (eGFR <15)	1 (0.1)
**Progression of kidney disease**[#], n (%)	Stable	460 (45.6)
	Progression	244 (24.2)
	Remission	304 (30.2)
**Number of comorbidities***, n (%)	None (CKD only)	56 (5.6)
	One	308 (30.6)
	Two	300 (29.8)
	Three or more	344 (34.1)
**Individual comorbidities***, n (%)	Hypertension	874 (86.7)
	Painful condition	300 (29.8)
	Anaemia	201 (19.9)
	Ischaemic heart disease	187 (18.6)
	Diabetes	143 (14.2)
	Thyroid disorder	127 (12.6)
	Cerebrovascular disease	96 (9.5)
	Chronic respiratory disorder	79 (7.8)
	Depression	59 (5.9)
	Peripheral vascular disease	29 (2.9)
	Heart failure	24 (2.4)
**Quality of Life domains** (Any	Mobility problems	582 (57.7)

For peer review only

For peer review only - http://bmjopen.bmj.com/site/about/guidelines.xhtml

problems reported in each EQ-5D-5L domain, n (%))	Self-care problems	166 (16.5)
	Usual activity problems	466 (46.2)
	Pain/discomfort	712 (70.6)
	Anxiety/depression	319 (31.6)
	No problems in any domain	191 (18.9)
**Functional status**# (KPS score), n (%)	Functional impairment (KPS ≤70)	234 (23.2)
	KPS >70 (able to carry on normal activity and to work; no special care needed)	774 (76.8)

Where variable category percentages sum to less than or more that 100%, this is due to rounding.
* Variables assessed at baseline. # Variables assessed at year five follow up.
** Includes Mixed, Asian, Cypriot and Other. Abbreviations SD= standard deviation, GCSE= General Certificate of Secondary Education, A level= Advanced level, NVQ= National Vocational Qualifications, IMD= Index of Multiple Deprivation, eGFR= estimated Glomerular Filtration Rate, uACR= Urine Albumin-to-Creatinine Ratio, IQR= Interquartile Range, KDIGO= Kidney Disease: Improving Global Outcomes, CKD= Chronic Kidney Disease, KPS= Karnofsky Performance Status

Approximately half (n=506, 50.3%) reported having had no formal education, just under half (n=497, 49.4%) lived in areas of lower deprivation (IMD quintiles four or five) and the majority (n=994, 98.6%) were white. The mean eGFR at follow-up was 54.0 ml/min/1.73m^2 (SD 15.2) and almost half (n=460, 45.6%) had had stable CKD over the preceding five-year period. Only 56 (5.6%) had no comorbidities and about a third (n=344, 34.1%) had three or more comorbidities. For comparison of basic characteristics of those with and without complete five year follow up data see supplementary Table S1. A slightly higher proportion of those with incomplete follow up data had three or more comorbidities and only a very small proportion had functional impairment (Table S1). The majority reported some impairment in HRQoL overall, with a median score of 75 out of 100 (interquartile range 60-90) on the EQ-VAS. A minority (n=378, 37.5%) had an EQ-5D-3L index score higher than the age/sex matched median, and only 18.9% of people (n=191) reported no problems across any of the individual HRQoL domains. Furthermore, a majority of participants reported some problems with mobility (n=582, 57.7%) and pain/discomfort (n=712, 70.6%) (Table 1).

When comparing the self-reported HRQoL domains with HSE data, the proportion of people in the RRID population reporting problems with mobility or pain/discomfort was higher (57.7% vs. 50.4%, and 70.6% vs. 60.1% respectively) than in the HSE population (Table 2).

Table 2. Comparison of the EQ-5D-5L quality of life domains between the Health Survey for England (HSE) 2012 and the Renal Risk in Derby (RRID) cohort

		HSE 2012 cohort* (N=258)		RRID CKD cohort (N=1008)	
		n	%	n	%
**Mobility**	1 (no problems in walking about)	128	49.6	426	42.3
	2 – 5 (some problems)	130	50.4	582	57.7
**Self-care**	1 (no problems washing or dressing)	209	81.0	842	83.5
	2 – 5 (some problems)	49	19.0	166	16.5

For peer review only - http://bmjopen.bmj.com/site/about/guidelines.xhtml

**Usual activities**	1 (no problems doing usual activities)	142	55.0	542	53.8
	2 – 5 (some problems)	116	45.0	466	46.2
**Pain/ discomfort**	1 (no pain or discomfort)	103	39.9	296	29.4
	2 – 5 (some pain or discomfort)	155	60.1	712	70.6
**Anxiety/ depression**	1 (not anxious or depressed)	188	72.9	689	68.4
	2 – 5 (some anxiety or depression)	70	27.1	319	31.6

* All participants were aged 65 years or above. CKD= Chronic Kidney Disease

For clinician-assessed FS, only two participants had performance status assessed as KPS ≤40 ('unable to care for self, requiring the equivalent of institutional or hospice care') and 232 (23%) were assessed as KPS 50-70 ('unable to work; able to live at home and care for most personal needs; varying amount of assistance needed').

The association between clinician-assessed FS and patient-reported HRQoL was complex, either when based on the index score (Figure 2a) or the VAS scale (Figure 2b). HRQoL was generally higher among those with better FS. However, the spread of HRQoL scores (using either of the HRQoL metrics) was broader among those with lower FS, suggesting a greater degree of variation in HRQoL among those with lower FS than among those with higher FS (Figure 2). A higher proportion of people with clinician-assessed functional impairment (KPS ≤70) reported having some degree of problems in each of the five EQ-5D-5L domains than people without functional impairment (Supplementary table S2).

Using the mobility domain as an example (Table 3), on univariable analysis older age, greater area deprivation level, higher number of comorbidities, poorer functional status, lower eGFR, higher level of albuminuria, lower educational attainment, and higher BMI were associated with having some problems.

Table 3. Logistic regression models examining associations between lower quality of life (EQ-5D-5L mobility domain categorised as 'no problems' vs. 'any problems') and patient characteristics. N=1008 in univariable models unless otherwise stated, N=1005 for final multivariable logistic regression models

		Univariable		Multivariable*	
		**OR (95% CI)**	**p**	**OR (95% CI)**	**p**
**Age (years)**		1.05 (1.03 – 1.07)	<0.001	1.03 (1.02 – 1.05)	0.001
**Female sex (vs. male)**		1.16 (0.89 – 1.49)	0.27	-	-
**Index of multiple deprivation (IMD quintile relative to England) (vs. quintile 5: least deprived)** [N=1006]	Quintile 1 (most deprived)	1.61 (0.96 – 2.69)	0.04#	0.95 (0.52 – 1.74)	0.436#
	Quintile 2	1.69 (1.17 – 2.43)		1.38 (0.90 – 2.10)	
	Quintile 3	1.10 (0.75 – 1.62)		0.94 (0.60 – 1.48)	
	Quintile 4	1.25 (0.88 – 1.78)		1.17 (0.78 – 1.76)	
**Number of comorbidities (vs. no comorbidities)**	One	1.25 (0.70 – 2.24)	<0.001#	1.01 (0.53 – 1.93)	0.002#
	Two	2.43 (1.35 – 4.38)		1.40 (0.72 – 2.71)	
	Three or more	4.50 (2.49 – 8.12)		2.10 (1.08 – 4.10)	
**Functional status (KPS score) (vs. KPS >70)**	Functional impairment (KPS ≤70)	26.03 (13.60 – 49.81)	<0.001	16.87 (8.70 – 32.79)	<0.001

For peer review only - http://bmjopen.bmj.com/site/about/guidelines.xhtml

**eGFR (ml/min/1.73m²)** [N=1007]		0.98 (0.98 – 0.99)	<0.001	0.99 (0.99 – 1.01)	0.983
**uACR (KDIGO categories) (vs. category A1, <3mg/mmol)** [N=1007]	A2 (3-29 mg/mmol)	1.39 (1.02 - 1.91)	0.074#	1.01 (0.69 – 1.48)	0.937#
	A3 (≥30mg/mmol)	1.42 (0.78 – 2.57)		0.88 (0.41 – 1.85)	
**Educational attainment (vs. first or higher degree or NVQ 4-5)** [N=1007]	No formal qualifications	2.20 (1.59 – 3.05)	<0.001#	1.38 (0.93 – 2.04)	0.238#
	GCSE, A level or NVQ 1-3	1.23 (0.86 – 1.76)		1.13 (0.75 – 1.70)	
**BMI (vs. <25 kg/m²))**	Overweight (BMI 25-29.99 kg/m²)	1.51 (1.07 – 2.13)	<0.001#	1.37 (0.93 – 2.01)	<0.001#
	Obese (BMI ≥30 kg/m²)	3.24 (2.26 – 4.63)		2.44 (1.61 – 3.69)	
**Smoking status (vs. never smoked)**	Current smoker	1.50 (0.79 – 2.87)	0.413#	-	-
	Ex-smoker	1.10 (0.85 – 1.42)		-	

* Adjusted for age, deprivation level, number of comorbidities, functional status, eGFR at five-year follow-up, uACR at five-year follow-up, educational attainment, and BMI. #p value for trend. Abbreviations: OR= Odds Ratio, CI= Confidence Interval, KPS= Karnofsky Performance Status, eGFR= estimated Glomerular Filtration Rate, uACR= Urinary Albumin to Creatinine Ratio, KDIGO= Kidney Disease Improving Global Outcomes, GCSE= General Certificate of Secondary Education, A level= Advanced level, NVQ= National Vocational Qualifications, BMI= Body Mass Index, IMD= Index of Multiple Deprivation.

In the fully adjusted multivariable model these associations remained for older age, higher number of comorbidities, poorer functional status, and higher BMI (Table 3). A summary of the main independent associations identified in the multivariable logistic regression models for usual activities, self-care, pain/discomfort and anxiety/depression are shown in Table 4 and the full analyses in Supplementary tables S3 to S6.

Table 4. Summary matrix of the independent associations with 'some problems' in each domain of the EQ-5D-5L (from multivariable logistic regression analyses)

	Mobility	Self care	Usual activities	Pain / discomfort	Anxiety / depression
**Increasing age**	○				
**Female sex**					○
**Greater area deprivation level**					
**Higher number of**	○	○	○	○	

For peer review only

**comorbidities**					
**Functional impairment**	○	○	○	○	○
**Lower eGFR**					
**Higher level of albuminuria**					
**Lower educational attainment**					○
**Higher BMI**	○	○	○	○	
**Smoking**		○			

Factors associated with a lower FS on univariable analysis included older age, lower socioeconomic status (assessed by both IMD score and educational attainment), higher number of comorbidities, obesity, reduced eGFR and greater degree of albuminuria. Other than reduced eGFR, all of these factors remained significant after adjustment (Table 5). No interactions were identified in any analyses.

For peer review only

For peer review only - http://bmjopen.bmj.com/site/about/guidelines.xhtml

Table 5. Logistic regression models of the associations between clinician-assessed functional impairment (KPS score ≤70) and patient characteristics. N=1008 in univariable models unless otherwise stated, N=1004 for final multivariable logistic regression model

		Univariable		Multivariable*	
		**OR (95% CI)**	**p**	**OR (95% CI)**	**p**
**Age (years)**		1.07 (1.05 – 1.09)	<0.001	1.07 (1.04 – 1.09)	<0.001
**Sex (vs. male)**		1.15 (0.85 – 1.56)	0.371	1.32 (0.91 – 1.91)	0.148
**Index of multiple deprivation** (IMD quintile relative to England) **(vs. quintile 5: least deprived)** [N=1006]	Quintile 1 (most deprived)	2.77 (1.55 – 4.95)	0.003[#]	2.03 (1.06 – 3.87)	0.045[#]
	Quintile 2	2.19 (1.39 – 3.44)		2.05 (1.24 – 3.40)	
	Quintile 3	1.83 (1.12 – 2.98)		2.02 (1.17 – 3.47)	
	Quintile 4	1.64 (1.03 – 2.59)		1.65 (1.00 – 2.75)	
**Number of comorbidities (vs. no comorbidities)**	One	1.18 (0.44 – 3.18)	<0.001[#]	0.62 (0.22 – 1.77)	<0.001[#]
	Two	3.10 (1.19 – 8.08)		1.22 (0.44 – 3.38)	
	Three or more	5.97 (2.32 – 15.35)		2.18 (0.80 – 5.96)	
**eGFR (ml/min/1.73m^2)** [N=1007]		0.97 (0.96 – 0.98)	<0.001	0.99 (0.98 – 1.00)	0.186
**uACR (KDIGO categories) (vs. category A1, <3mg/mmol)** [N=1007]	A2 (3-29mg/mmol)	1.92 (1.37 - 2.70)	0.002[#]	1.92 (1.29 – 2.87)	0.005[#]
	A3 (≥30mg/mmol)	1.96 (1.05 – 3.66)		1.74 (0.82 – 3.68)	
**Educational attainment (vs. first or higher degree or NVQ 4-5)** [N=1007]	No formal qualifications	2.77 (1.81 – 4.26)	<0.001[#]	2.08 (1.26 – 3.41)	<0.001[#]
	GCSE, A level or NVQ 1-3	1.07 (0.65 – 1.77)		0.99 (0.56 – 1.75)	
**BMI (vs. <25 kg/m^2)**	Overweight (BMI 25- 29.99 kg/m^2)	1.54 (0.94 – 2.53)	<0.001[#]	1.59 (0.93 – 2.73)	<0.001[#]
	Obese (BMI ≥30kg/m^2)	3.76 (2.34 – 6.04)		4.23 (2.48 – 7.20)	
**Smoking status (vs. never smoked)**	Current smoker	1.37 (0.70 – 2.71)	0.563[#]	-	-
	Ex-smoker	0.95 (0.70 – 1.28)		-	

* Adjusted for age, sex, deprivation level, number of comorbidities, eGFR at five-year follow-up, ACR, educational attainment and BMI. [#]P value for trend. Abbreviations: OR= Odds Ratio, CI= Confidence Interval, eGFR= Estimated Glomerular Filtration Rate, ACR= Albumin Creatinine Ratio, KDIGO= Kidney Disease Improving Global Outcomes, GCSE= General Certificate of Secondary Education, A level= Advanced level, NVQ= National Vocational Qualifications, BMI= Body Mass Index, IMD= Index of Multiple Deprivation.

For peer review only

For peer review only - http://bmjopen.bmj.com/site/about/guidelines.xhtml

DISCUSSION

In this cross-sectional study of people with mild to moderate CKD who survived to year five in a UK primary care cohort, overall patient-reported HRQoL was relatively high though a substantial proportion of participants reported problems in each HRQoL domain. A majority reported problems with mobility and pain/discomfort. Although most people had a clinician-assessed FS suggesting that they were able to carry on normal activity and to work with no special care needed, about a quarter were assessed as having functional impairment (being unable to work but able to live at home and care for most personal needs with a varying amount of assistance needed). HRQoL was generally higher among those with better FS but there was more variation in HRQoL among those with lower FS, and low FS was independently strongly associated with low HRQoL in regression analyses. Higher number of comorbidities and obesity were independently associated with problems in most EQ-5D-5L domains and with functional impairment. Functional impairment was independently associated with experiencing some problems across all EQ-5D-5L domains.

This study had several strengths, including the large size of the cohort, and recruitment from primary care, a setting in which patients with mild to moderate CKD are typically managed. The RRID cohort is pragmatic and likely to represent a population of typical patients with mild to moderate CKD in the UK.[22,23] We were able to identify a broad range of comorbidities but they were identified at baseline only. The number of comorbidities may therefore have changed by the time of follow-up assessment, meaning that our comorbidity prevalence data were likely underestimates of the true prevalence in some patients. Similarly certain other exposures were assessed at baseline and could potentially have changed by the time of follow-up. We recognise these as important limitations but consider that they are unlikely to significantly alter the main findings of our study with regard to HRQoL and FS. A further strength is that we were able to measure HRQoL and FS in the same patient group and the HRQoL and FS data were relatively complete. The use of the EQ-5D-5L index measure and data from the HSE enabled comparison with a general population. However, the index values for HRQoL required conversion to 3L values as reliable 5L index values are not yet available for all standard populations. Evidence from a previous RRID analysis on prior renal function change provided depth to our analyses for this cross-sectional study. However, there were also several important limitations – this was a cross-sectional study of survivors and we were therefore not able to draw causative links. HRQoL and FS measures were taken at five-year follow-up but not at baseline and we were therefore unable to identify change over time. The RRID cohort is predominantly comprised of people of white ethnicity, limiting generalisability of our findings. Comparison with HSE data was undertaken only via univariable analyses, such that potential confounding factors may have influenced the differences observed between the two groups. We also did not have sufficient numbers to allow for reliable exploration of associations between specific comorbidities and HRQoL or FS. We also recognise the need for caution in the interpretation of the associations between functional impairment and problems in individual domains due to small numbers in some individual categories (leading to wide confidence intervals). A further limitation is that one inclusion criterion was the ability to attend study visits, which would have resulted in some selection bias by excluding the very frail.

People with CKD are likely to have multiple comorbidities due both to the nature of the disease process and the relationship between CKD and older age. We have identified that comorbidity count was an independent determinant of both HRQoL and FS, highlighting the importance of a holistic approach that includes attention to comorbidities in the management of people with mild to moderate CKD. As reported previously, 40% of people in this cohort with stage 3 disease had at least two comorbidities.[6] There are clearly shared risk factors for several of the included comorbidities. It is therefore perhaps unsurprising that in a large cohort of over half a million Canadian patients with

For peer review only - http://bmjopen.bmj.com/site/about/guidelines.xhtml

CKD, comorbidities such as hypertension and diabetes were common (46.6% and 17.8% respectively). However, a substantial number of patients also had ‘discordant’ comorbidities such as chronic pulmonary disease (14.0%) and 10.6% of patients had chronic pain.[5] Comorbidities were all associated with an increased risk of hospitalisation.[5] It is striking that the majority of people in our cohort reported problems with mobility and chronic pain/discomfort, and that both were more prevalent than in a nationally-representative sample of the English general population of similar age. About 30% of our cohort were taking analgesic medication, but about 71% reported pain or discomfort in the EQ-5D-5L. This likely reflects the association of CKD with comorbidities, since mild to moderate reductions in GFR are unlikely to cause poor mobility or pain. Nevertheless, this observation further highlights the need to pay attention to mobility issues and pain management in order to improve quality of life in people with stage 3 CKD.

The prevalence of diabetes in this population of people with CKD stage 3 was 14.2%. This is lower than the prevalence of 20.1% noted in analyses of CKD prevalence in the Health Survey for England.[2] It is possible that this relates to this study population comprising survivors at five years in a cohort study and those with diabetes may have been more likely to die prior to these analyses than those without. The study population was also predominantly white and those ethnic groups with greater diabetes prevalence were therefore under-represented.

Mental health problems are common with 26% of adults in England reporting a diagnosis at some point in time.[24] In our study, about 6% of people were classified as having a depressive condition, defined pragmatically based on current antidepressant use or patient self-report, although about 32% reported some anxiety or depression in the EQ-5D-5L, so this was probably an underestimate. In a large meta-analysis, approximately 25% of adults with CKD stage 1-5 had symptoms suggestive of depression.[25] This appears to persist even in milder forms of the disease and a large study from 2012 showed the prevalence of depression in people with CKD whose eGFR was ≥60 ml/min/1.73m^2 was 23.6%.[26] These data imply that careful attention to mental health problems, including screening for depression, may also be key interventions to improve HRQoL in people with mild to moderate CKD.

Being able to carry out activities of daily living (ADLs) is an important part of living independently. There is a recognised association between having difficulty with ADLs and a lower HRQoL in older adults, though comparison of HRQoL (as assessed by EQ-5D-5L) with FS (as assessed by the KPS) is under-studied.[27] The strong association observed in this study between functional impairment and lower HRQoL including mobility, self care and usual actitivies, even after adjustment for potential confounding factors, suggests that clinicians identifying functional impairment should consider their patient’s HRQoL. Previous research has identified higher prevalence of problems with ADLs among people with CKD (defined as eGFR<60ml/min/1.73m^2) than people without, variously reported as between 26% and 55% depending on the nature of the population studied and the measure of ADL used.[28-30] Two large longitudinal studies have shown, in keeping with our findings, a significant reduction in instrumental ADLs and basic ADLs when renal function deteriorates over time.[28,29] It should also be noted that the KPS is a clinician-assessed tool comprising an element of subjectivity and therefore may be less accurate than an in-depth questionnaire looking at both instrumental ADLs and generic ADLs.

In 2016, NICE published their first guideline for the management of individuals with multiple co-existing chronic diseases.[31] These guidelines ask clinicians to consider the overall burdens of disease and treatment, and to discuss these with patients and develop individualised management plans. Amongst other things, they suggest that clinicians assess for frailty (using measures such as gait speed or self-reported health status) and remain particularly vigilant for issues with mental health or chronic pain.[31] Our data provide evidence that a similar approach is warranted in people with mild to moderate CKD. When managing these patients, clinicians should consider comorbidities and discuss how treatments will fit in with those for other comorbidities, particularly mental health and chronic pain-related conditions and those affecting mobility. Obesity, as a

For peer review only - http://bmjopen.bmj.com/site/about/guidelines.xhtml

potentially modifiable factor with an independent association with both functional impairment and HRQoL in our study, is also an important consideration. This could include devising management plans jointly with other healthcare professionals, streamlining health services to cater for people with multiple conditions, and signposting to appropriate services. Both General Practitioners and nephrologists should be well-placed to manage patients holistically, but patients would benefit from mental health, comorbidity and pain issues being considered in clinical guidelines and outcome measures. It is also important to consider the capacity of people to self-manage, and the extent of patient activation, if mental health issues or multiple comorbidities are present.

CONCLUSION

We have observed that people with mild to moderate CKD commonly have multiple comorbidities and many report some HRQoL problems and functional impairment. Taken together with previous observations that the risk of CKD progression is low in this context, these data suggest that mild to moderate CKD in older people could be more important as a marker of increased comorbidity than as a risk factor for ESKD. Clinicians should therefore carefully consider comorbidities and discuss how treatments will fit in with those for other comorbidities, particularly mental health, chronic pain-related conditions and those affecting functional status.

For peer review only

For peer review only - http://bmjopen.bmj.com/site/about/guidelines.xhtml

Competing interest statement
None of the authors have any conflicts of interest to declare**.** The results presented in this paper have not been published previously in whole or part, except in abstract format.

Author contributions
SDSF, JB, PJR, JEM and HMY designed and undertook the analyses. AS and MWT obtained the cohort five year follow up data. MWT, NJM, RJF, and CWM established the RRID cohort, provided data and contributed development of the project and critically reviewed the manuscript. All authors critically reviewed the paper; were involved in the drafting and approval of the final manuscript; and act as guarantors. All authors take responsibility for the data and research governance.

Acknowledgements
The authors acknowledge the contributions of the participants, their families and the participating general practice surgeries without whom this work would not have been possible. The authors would like to thank Shihua Zu who provided advice on using EQ-5D-5L.

Funding
The RRID study was funded by a Research Project Grant (R302/0713) from the Dunhill Medical Trust (http://www.dunhillmedical.org.uk). Previous study funding includes a joint British Renal Society (http://www.britishrenal.org) and Kidney Research UK (http://www.kidneyresearchuk.org) fellowship (BRS3/2008, to NJM), and an unrestricted educational grant (EPWE124712-G) from Roche Products Ltd. (https://www.roche.co.uk). The funders had no role in study design, data collection and analysis, decision to publish, or preparation of the manuscript.

Ethics approval
The RRID study was approved by the Nottingham Research Ethics Committee 1 and was included on the National Institute for Health Research (NIHR) Clinical Research Portfolio (NIHR Study ID: 6632). All participants provided written, informed consent. The RRID study complies with the Declaration of Helsinki and the principles of Good Clinical Practice.

Data sharing statement
Anonymised data can be made available to researchers who meet the conditions of the ethics approval and research governance policy that applies to this study. Researchers may apply for data access by contacting Dr. Teresa Grieve, Research and Development Manager, University Hospitals of Derby and Burton NHS Foundation Trust (teresa.grieve@nhs.net).

Transparency statement
We affirm that the manuscript is an honest, accurate, and transparent account of the study being reported. No important aspects of the study have been omitted and any discrepancies from the study as originally planned have been explained.

For peer review only - http://bmjopen.bmj.com/site/about/guidelines.xhtml

Figure legends

Figure 1. Flow chart of study participants

Figure 2. Relationship between quality of life and functional status

2a. Functional status (by Karnofsky score) and quality of life (by EQ-5D-3L Index score)
2b. Functional status (by Karnofsky score) and quality of life (by EQ-5D self-reported VAS scale)

For peer review only

For peer review only - http://bmjopen.bmj.com/site/about/guidelines.xhtml

REFERENCES

1. Hill NR, Fatoba ST, Oke JL, et al. Global Prevalence of Chronic Kidney Disease – A Systematic Review and Meta-Analysis. *PLoS One* 2016; 11(7): e0158765
2. Fraser SDS, Aitken GR, Taal MW, et al. Exploration of chronic kidney disease prevalence estimates using new measures of kidney function in the Health Survey for England. *PLoS One* 2015; 10(2): e0118676
3. NICE Clinical Guidelines 182. Chronic Kidney Disease: Early Identification and Management of Chronic Kidney Disease in Adults in Primary and Secondary Care. London: National Institute of Health and Care Excellence; 2014.
4. Shardlow A, McIntyre NJ, Fluck RJ, McIntyre CW, Taal MW. Chronic Kidney Disease in Primary Care: Outcomes after Five Years in a Prospective Cohort Study. *PLoS Med* 2016;13(9):e1002128
5. Tonelli M, Wiebe N, Guthrie B, et al. Comorbidity as a driver of adverse outcomes in people with chronic kidney disease. *Kidney Int* 2015;88:859-866.
6. Fraser SDS, Roderick PJ, May CR, et al. The burden of comorbidity in people with chronic kidney disease stage 3: a cohort study. *BMC Nephrol* 2015;16:193.
7. Jesky MD, Dutton M, Dasgupta I, et al. Health-Related Quality of Life Impacts Mortality but Not Progression to End-Stage Renal Disease in Pre-Dialysis Chronic Kidney Disease: A Prospective Observational Study. *PLoS One* 2016;11(11):e0165675.
8. Roberti J, Cummings A, Myall M, et al. Work of being an adult patient with chronic kidney disease: a systematic review of qualitative studies. BMJ Open 2018;8(9):e023507.
9. McIntyre N, Fluck R, McIntyre CW, Taal MW. Risk profile in chronic kidney disease stage 3: older versus younger patients. *Nephron Clin Pract* 2011;119(4):269- 276.
10. Herdman M, Gudex C, Lloyd A, Janssen MF, Kind P, Parkin D. Development and preliminary testing of the new five-level version of EQ-5D (EQ-5D-5L). *Qual Life Res* 2011; 20(10):1727-1736.
11. Devlin N, Shah K, Feng Y, Mulhern B, van Hout B. Valuing health-related quality of Life: An EQ-5D-5L Value Set for England. *Health Econ* 2018.27(1):7-22.
12. NICE position statement on use of the EQ-5D-5L value set for England. National Institute of Health and Care Excellence (updated October 2019). https://www.nice.org.uk/about/what-we-do/our-programmes/nice-guidance/technology-appraisal-guidance/eq-5d-5l. Accessed 26th March 2020
13. EuroQol EQ-5D-5L Crosswalk Index Value Calculator. https://euroqol.org/eq-5d-instruments/eq-5d-5l-about/valuation-standard-value-sets/crosswalk-index-value-calculator/. Accessed 26th March 2020
14. van Hout B, Janssen MF, Feng YS, et al. Interim scoring for the EQ-5D-5L: mapping the EQ-5D-5L to EQ-5D-3L value sets. *Value Health*. 2012:15(5):708-15
15. Karnofsky D, Abelmann W, Craver L, Burchenal J. The use of nitrogen mustards in the palliative treatment of cancer. *Cancer* 1948, 1: 634-656.
16. Salloum RG, Smith TJ, Jensen GA, et al. Using Claims-Based Measures to Predict Performance Status Score in Lung Cancer Patients *Cancer* 2011;117(5):1038- 48
17. KDIGO 2012 clinical practice guideline for the evaluation and management of chronic kidney disease. *Kidney Int Suppl*. 2013;3(1):150.
18. Pinto-Sietsma S, Navis G, Janssen W et al. A central body fat distribution is related to renal function impairment, even in lean subjects. *Am J Kidney Dis*. 2003;41:733-741
19. Jordan H, Roderick P, Martin D. The index of multiple deprivation 2000 and accessibility effects on health. *J Epidemiol Community Health*. 2004;58:250-7.

For peer review only

20. Feng Y, Devlin N, Herdman M. Assessing the health of the general population in England: how do the three- and five-level versions of EQ-5D compare? *Health Qual Life Outcomes* 2015;13:171
21. Breeze E, Jones DA, Wilkinson P et al. Area deprivation, social class, and quality of life among people aged 75 years and over in Britain. *Int J Epidemiol*. 2005;34(2):276-83.
22. de Lusignan SD, Gallagher H, Jones S et al. Audit-based education lowers systolic blood pressure in chronic kidney disease: the Quality Improvement in CKD (QICKD) trial results. *Kidney Int*. 2013;84(3):609-20.
23. Major RW, Brown C, Shepherd D et al. The Primary-Secondary Care Partnership to Improve Outcomes in Chronic Kidney Disease (PSP-CKD) Study: A Cluster Randomized Trial in Primary Care. *J Am Soc Nephrol* 2019;30(7):1261-1270.
24. Bridges S. Mental Health Problems. Health Survey for England 2014. Vol 1 Ch 2. The Health and Social Care Information Centre 2015.
25. Palmer S, Vecchio M, Craig J, et al. Prevalence of depression in chronic kidney disease : systematic review and meta-analysis of observational studies. *Kidney Int* 2013;84(1):179-91.
26. Fischer MJ, Xie D, Jordan N et al. CRIC Study Group Investigators. Factors associated with depressive symptoms and use of antidepressant medications among participants in the Chronic Renal Insufficiency Cohort (CRIC) and Hispanic-CRIC Studies. *Am J Kidney Dis*. 2012 ;60(1):27-38.
27. Lyu W, Wolinsky F. The onset of ADL difficulties and changes in health-related quality of life. *Health Qual Life Outcomes* 2017;15(1):217.
28. Bowling C, Sawyer P, Campbell R, Ahmed A, Allman RM. Impact of chronic kidney disease on activities of daily living in community-dwelling older adults. *J Gerontol A Biol Sci Med Sci* 2011;66(6):689-94.
29. Chin H, Ahn S, Ryu J et al. Renal function and decline in functional capacity in older adults. *Age Ageing* 2014;43:833-8.
30. Smyth A, Glynn L, Murphy A. Mild chronic kidney disease and functional impairment in community-dwelling older adults. *Age Ageing* 2013;42:488-94.
31. National Institute for Health and Care Excellence (NICE). Multimorbidity: clinical assessment and management (NG56). Sept 2016.

For peer review only

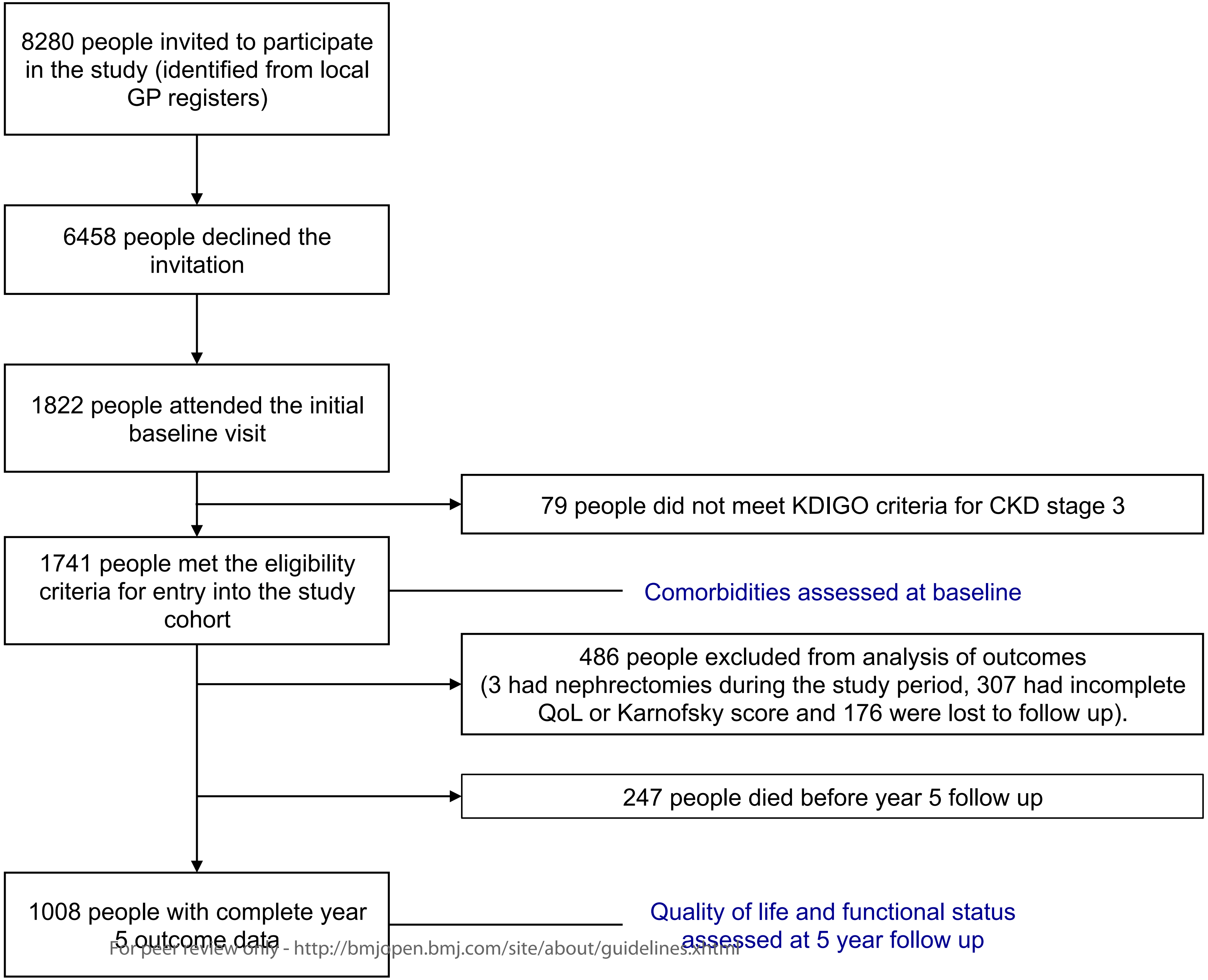
8280 people invited to participate in the study (identified from local GP registers)
6458 people declined the invitation
1822 people attended the initial baseline visit
79 people did not meet KDIGO criteria for CKD stage 3
1741 people met the eligibility criteria for entry into the study cohort
Comorbidities assessed at baseline
486 people excluded from analysis of outcomes (3 had nephrectomies during the study period, 307 had incomplete QoL or Karnofsky score and 176 were lost to follow up).
247 people died before year 5 follow up
1008 people with complete year 5 outcome data
Quality of life and functional status assessed at 5 year follow up

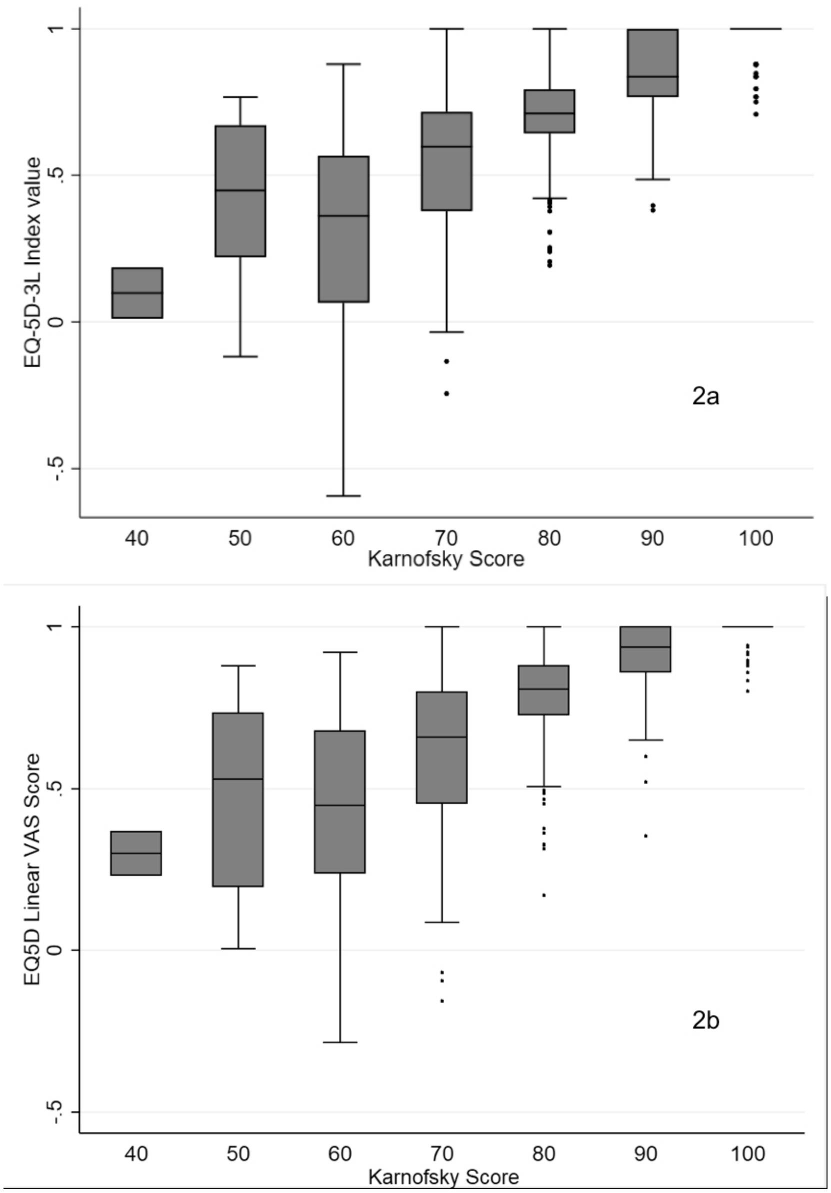

Figure 2. Relationship between quality of life and functional status

2a. Functional status (by Karnofsky score) and quality of life (by EQ-5D-3L Index score)
2b. Functional status (by Karnofsky score) and quality of life (by EQ-5D self-reported VAS scale)

72x105mm (300 x 300 DPI)

Supplementary material

Table S1. Characteristics of those with and without complete follow up data at five years.

	Complete HRQoL data (n=1008)	Incomplete HRQoL data (n=486)
**Age (mean(SD))**	70.6 (8.6)	74.7 (9.0)
**Sex (n(%) male)**	387 (38)	160 (33)
**Index of multiple deprivation (IMD) (n (%) in most deprived quintile)**	82 (8)	49 (10)
**Comorbidities (n (%) with three or more)**	344 (34)	206 (42)
**Functional impairment (KPS ≤70)**	234 (23)	2 (0.4%)

Table S2. Associations between patient-reported EQ-5D-5L quality of life domains and clinician-assessed functional status.

Patient-reported EQ-5D-5L quality of life domain		No clinician-assessed functional impairment (KPS score >70)		Clinician-assessed functional impairment (KPS score ≤70)		Total RRID cohort		
		n	%*	n	%*	n	%*	p**
**Mobility**	1 (no problems in walking about)	416	53.8	10	4.3	426	42.3	<0.001
	2 – 5 (some problems)	358	46.2	224	95.7	582	57.7	
**Self care**	1 (no problems washing or dressing)	726	93.8	116	49.6	842	83.5	<0.001
	2 – 5 (some problems)	48	6.2	118	50.4	166	16.5	
**Usual activities**	1 (no problems doing usual activities)	508	65.6	34	14.5	542	53.8	<0.001
	2 – 5 (some problems)	266	34.4	200	85.5	466	46.2	
**Pain/ discomfort**	1 (no pain or discomfort)	270	34.9	26	11.1	296	29.4	<0.001
	2 – 5 (some pain or discomfort)	504	65.1	208	88.9	712	70.6	
**Anxiety/ depression**	1 (not anxious or depressed)	576	74.4	113	48.3	689	68.4	<0.001
	2 – 5 (some anxiety or depression)	198	25.6	121	51.7	319	31.6	
**EQ-5D-3L index score (converted from EQ-5D-5L)**	> median for age and sex	351	45.4	27	11.5	378	37.5	<0.001
	≤ median for age and sex	423	54.7	207	88.5	630	62.5	
**Total**		774	100.0	234	100.0	1008	100.0	

For peer review only - http://bmjopen.bmj.com/site/about/guidelines.xhtml

* Column percentages are shown.** Chi-square test was performed. Abbreviations: KPS= Karnofsky Performance Status, RRID= Renal Risk in Derby

Tables S3 to S6. Logistic regression models examining associations between lower quality of life (EQ-5D-5L domains of usual activities, self-care, pain/discomfort and anxiety/depression categorised as ‘no problems’ vs. ‘any problems’) and patient characteristics. N=1008 in univariable models unless otherwise stated, N=1005 for final multivariable logistic regression models

S3. USUAL ACTIVITIES

		Univariable		Multivariable*	
		**OR (95% CI)**	**p**	**OR (95% CI)**	**p**
**Age (years)**		1.03 (1.02 – 1.05)	<0.001	1.02 (1.00 – 1.04)	0.056
**Female sex (vs. male)**		1.05 (0.81 – 1.36)	0.71	-	-
**Index of multiple deprivation (IMD quintile relative to England) (vs. quintile 5: least deprived) [N=1006]**	Quintile 1 (most deprived)	1.45 (0.87 – 2.40)	0.009#	0.83 (0.46 – 1.52)	0.076#
	Quintile 2	1.93 (1.34 – 2.78)		1.64 (1.08 – 2.50)	
	Quintile 3	1.43 (0.97 – 2.12)		1.37 (0.87 – 2.15)	
	Quintile 4	1.25 (0.87 – 1.78)		1.12 (0.74 – 1.69)	
**Number of comorbidities (vs. no comorbidities)**	One	1. 50 (0.77 – 2.91)	<0.001#	1.41 (0.68 – 2.90)	<0.001#
	Two	2.56 (1.32 – 4.97)		1.82 (0.88 – 3.78)	
	Three or more	6.34 (3.28 – 12.25)		4.20 (2.02 – 8.74)	
**Functional status (KPS score) (vs. KPS >70)**	Functional impairment (KPS ≤70)	11.23 (7.59 – 16.64)	<0.001	8.27 (5.43 – 12.58)	<0.001
**eGFR (ml/min/1.73m²) [N=1007]**		0.99 (0.98 – 1.00)	0.013	1.01 (1.00 – 1.02)	0.139
**uACR (KDIGO categories) (vs. category A1, <3mg/mmol) [N=1007]**	A2 (3-29 mg/mmol)	1.24 (0.92 - 1.68)	0.090#	0.92 (0.63 – 1.34)	0.767#
	A3 (≥30mg/mmol)	1.72 (0.97 – 3.09)		1.19 (0.59 – 2.43)	
**Educational attainment (vs. first or higher degree or NVQ 4-5) [N=1007]**	No formal qualifications	1.88 (1.35 – 2.62)	<0.001#	1.22 (0.82 – 1.82)	0.549#
	GCSE, A level or NVQ 1-3	1.30 (0.90 – 1.87)		1.24 (0.82 – 1.89)	
**BMI (vs. <25 kg/m²))**	Overweight (BMI 25-29.99 kg/m²)	1.54 (1.08 – 2.21)	<0.001#	1.38 (0.92 – 2.06)	0.019#
	Obese (BMI ≥30 kg/m²)	2.81 (1.96 – 4.03)		1.82 (1.19 – 2.76)	
**Smoking status (vs. never smoked)**	Current smoker	0.85 (0.46 – 1.60)	0.421#	-	-
	Ex-smoker	1.15 (0.89 – 1.49)		-	

*Adjusted for age, deprivation level, number of comorbidities, functional status, eGFR at five-year follow-up, uACR at five-year follow-up, educational attainment, and BMI. #p value for trend. Abbreviations: OR= Odds Ratio, CI= Confidence Interval, KPS= Karnofsky Performance Status, eGFR= estimated Glomerular Filtration Rate, uACR= Urinary Albumin to Creatinine Ratio, KDIGO= Kidney Disease Improving Global Outcomes,

For peer review only - http://bmjopen.bmj.com/site/about/guidelines.xhtml

GCSE= General Certificate of Secondary Education, A level= Advanced level, NVQ= National Vocational Qualifications, BMI= Body Mass Index, IMD= Index of Multiple Deprivation.

S4. SELF CARE

		Univariable		Multivariable*	
		OR (95% CI)	p	OR (95% CI)	p
**Age (years)**		1.01 (0.99 – 1.03)	0.472	-	-
**Female sex (vs. male)**		0.91 (0.64 – 1.27)	0.568	-	-
**Index of multiple deprivation (IMD quintile relative to England) (vs. quintile 5: least deprived) [N=1006]**	Quintile 1 (most deprived)	2.27 (1.19 – 4.34)	0.041[#]	1.12 (0.51 – 2.42)	0.895[#]
	Quintile 2	1.90 (1.15 – 3.15)		1.20 (0.67 – 2.19)	
	Quintile 3	1.64 (0.95 – 2.84)		1.31 (0.68 – 2.51)	
	Quintile 4	1.26 (0.75 – 2.13)		0.99 (0.54 – 1.85)	
**Number of comorbidities (vs. no comorbidities)**	One	1.43 (0.41 – 4.92)	<0.001[#]	1.27 (0.34 – 4.84)	0.050[#]
	Two	3.37 (1.01 – 11.21)		1.89 (0.51 – 6.97)	
	Three or more	6.45 (1.97 – 21.15)		2.64 (0.72 – 9.67)	
**Functional status (KPS score) (vs. KPS >70)**	Functional impairment (KPS ≤70)	15.39 (10.43 – 22.69)	<0.001	13.08 (8.46 – 20.22)	<0.001
**eGFR (ml/min/1.73m^2) [N=1007]**		0.99 (0.98 – 1.00)	0.055	1.01 (0.99 – 1.21)	0.154
**uACR (KDIGO categories) (vs. category A1, <3mg/mmol) [N=1007]**	A2 (3-29 mg/mmol)	1.37 (0.93 - 2.03)	0.223 [#]	-	-
	A3 (≥30mg/mmol)	1.39 (0.67 – 2.86)		-	
**Educational attainment (vs. first or higher degree or NVQ 4-5) [N=1007]**	No formal qualifications	1.91 (1.18 – 3.08)	0.014[#]	1.11 (0.63 – 1.97)	0.669[#]
	GCSE, A level or NVQ 1-3	1.31 (0.76 – 2.23)		1.32 (0.69 – 2.50)	
**BMI (vs. <25 kg/m^2))**	Overweight (BMI 25-29.99 kg/m^2)	1.20 (0.68 – 2.13)	<0.001[#]	0.95 (0.49 – 1.82)	0.003[#]
	Obese (BMI ≥30 kg/m^2)	3.47 (2.03 – 5.92)		1.98 (1.06 – 3.71)	
**Smoking status (vs. never smoked)**	Current smoker	2.17 (1.05 – 4.51)	0.018[#]	2.53 (1.00 – 6.40)	0.006[#]
	Ex-smoker	1.53 (1.08 – 2.16)		1.89 (1.24 – 2.88)	

* Adjusted for deprivation level, number of comorbidities, functional status, eGFR at five-year follow-up, educational attainment, BMI and smoking. [#]p value for trend. Abbreviations: OR= Odds Ratio, CI= Confidence Interval, KPS= Karnofsky Performance Status, eGFR= estimated Glomerular Filtration Rate, uACR= Urinary Albumin to Creatinine Ratio, KDIGO= Kidney Disease Improving Global Outcomes, GCSE= General Certificate of Secondary Education, A level= Advanced level, NVQ= National Vocational Qualifications, BMI= Body Mass Index, IMD= Index of Multiple Deprivation.

For peer review only

For peer review only - http://bmjopen.bmj.com/site/about/guidelines.xhtml

S5. PAIN / DISCOMFORT

		Univariable		Multivariable*	
		OR (95% CI)	p	OR (95% CI)	p
**Age (years)**		1.02 (1.00 – 1.03)	0.026	1.01 (0.99 – 1.02)	0.550
**Female sex (vs. male)**		1.23 (0.93 – 1.63)	0.141	-	-
**Index of multiple deprivation (IMD quintile relative to England) (vs. quintile 5: least deprived) [N=1006]**	Quintile 1 (most deprived)	2.42 (1.30 – 4.50)	0.046#	1.87 (0.97 – 3.60)	0.381#
	Quintile 2	1.52 (1.03 – 2.24)		1.33 (0.88 – 2.03)	
	Quintile 3	1.27 (0.84 – 1.92)		1.26 (0.81 – 1.95)	
	Quintile 4	1.25 (0.86 – 1.82)		1.24 (0.83 – 1.84)	
**Number of comorbidities (vs. no comorbidities)**	One	1.62 (0.91 – 2.86)	<0.001#	1.49 (0.82 – 2.71)	<0.001#
	Two	2.95 (1.65 – 5.29)		2.27 (1.22 – 4.20)	
	Three or more	4.70 (2.60 – 8.48)		3.06 (1.63 – 5.73)	
**Functional status (KPS score) (vs. KPS >70)**	Functional impairment (KPS ≤70)	4.29 (2.78 – 6.61)	<0.001	2.94 (1.86 – 4.67)	<0.001
**eGFR (ml/min/1.73m^2) [N=1007]**		0.99 (0.98 – 1.00)	0.150	-	-
**uACR (KDIGO categories) (vs. category A1, <3mg/mmol) [N=1007]**	A2 (3-29 mg/mmol)	0.87 (0.62 - 1.20)	0.670#	-	-
	A3 (≥30mg/mmol)	1.04 (0.55 – 1.96)		-	
**Educational attainment (vs. first or higher degree or NVQ 4-5) [N=1007]**	No formal qualifications	1.67 (1.19 – 2.35)	0.007#	1.23 (0.84 – 1.97)	0.075#
	GCSE, A level or NVQ 1-3	1.65 (1.13 – 2.41)		1.59 (1.06 – 2.38)	
**BMI (vs. <25 kg/m^2))**	Overweight (BMI 25-29.99 kg/m^2)	1.63 (1.15 – 2.30)	<0.001#	1.47 (1.02 – 2.12)	<0.001#
	Obese (BMI ≥30 kg/m^2)	3.26 (2.23 – 4.76)		2.37 (1.58 – 3.55)	
**Smoking status (vs. never smoked)**	Current smoker	1.12 (0.56 – 2.24)	0.945#	-	-
	Ex-smoker	1.02 (0.77 – 1.34)		-	

Adjusted for age, deprivation level, number of comorbidities, functional status, educational attainment, and BMI. #p value for trend. Abbreviations: OR= Odds Ratio, CI= Confidence Interval, KPS= Karnofsky Performance Status, eGFR= estimated Glomerular Filtration Rate, uACR= Urinary Albumin to Creatinine Ratio, KDIGO= Kidney Disease Improving Global Outcomes, GCSE= General Certificate of Secondary Education, A level= Advanced level, NVQ= National Vocational Qualifications, BMI= Body Mass Index, IMD= Index of Multiple Deprivation.

For peer review only

S6. ANXIETY / DEPRESSION

		Univariable		Multivariable*	
		**OR (95% CI)**	**p**	**OR (95% CI)**	**p**
**Age (years)**		1.00 (0.98 – 1.01)	0.942	-	-
**Female sex (vs. male)**		1.63 (1.23 – 2.16)	0.001	1.60 (1.18 –2.16)	0.002
**Index of multiple deprivation (IMD quintile relative to England) (vs. quintile 5: least deprived) [N=1006]**	Quintile 1 (most deprived)	0.89 (0.51 – 1.54)	0.856[#]	-	-
	Quintile 2	0.94 (0.64 – 1.38)		-	
	Quintile 3	1.14 (0.76 – 1.72)		-	
	Quintile 4	0.95 (0.65 – 1.38)		-	
**Number of comorbidities (vs. no comorbidities)**	One	0.80 (0.43 – 1.51)	0.005[#]	0.94 (0.49 – 1.81)	0.269[#]
	Two	1.25 (0.67 – 2.34)		1.29 (0.67 – 2.47)	
	Three or more	1.48 (0.80 – 2.75)		1.30 (0.68 – 2.49)	
**Functional status (KPS score) (vs. KPS >70)**	Functional impairment (KPS ≤70)	3.12 (2.30 – 4.22)	<0.001	3.08 (2.23 – 4.27)	<0.001
**eGFR (ml/min/1.73m^2) [N=1007]**		1.00 (0.99 – 1.01)	0.998	-	-
**uACR (KDIGO categories) (vs. category A1, <3mg/mmol) [N=1007]**	A2 (3-29 mg/mmol)	1.00 (0.72 - 1.38)	0.851[#]	-	-
	A3 (≥30mg/mmol)	0.83 (0.44 – 1.57)		-	
**Educational attainment (vs. first or higher degree or NVQ 4-5) [N=1007]**	No formal qualifications	1.51 (1.04 – 2.18)	0.009[#]	1.05 (0.71 – 1.56)	0.009[#]
	GCSE, A level or NVQ 1-3	1.86 (1.25 – 2.77)		1.67 (1.10 – 2.52)	
**BMI (vs. <25 kg/m^2))**	Overweight (BMI 25-29.99 kg/m^2)	0.94 (0.65 – 1.36)	0.133[#]	-	-
	Obese (BMI ≥30 kg/m^2)	1.26 (0.87 – 1.82)		-	
**Smoking status (vs. never smoked)**	Current smoker	1.00 (0.51 – 1.94)	0.989[#]	-	-
	Ex-smoker	0.98 (0.75 – 1.29)		-	

* Adjusted for sex, number of comorbidities, and functional status. [#]p value for trend. Abbreviations: OR= Odds Ratio, CI= Confidence Interval, KPS= Karnofsky Performance Status, eGFR= estimated Glomerular Filtration Rate, uACR= Urinary Albumin to Creatinine Ratio, KDIGO= Kidney Disease Improving Global Outcomes, GCSE= General Certificate of Secondary Education, A level= Advanced level, NVQ= National Vocational Qualifications, BMI= Body Mass Index, IMD= Index of Multiple Deprivation.

For peer review only

For peer review only - http://bmjopen.bmj.com/site/about/guidelines.xhtml

STROBE 2007 (v4) checklist of items to be included in reports of observational studies in epidemiology*

Checklist for cohort, case-control, and cross-sectional studies (combined)

Section/Topic	Item #	Recommendation	Reported on page #
**Title and abstract**	1	(*a*) Indicate the study's design with a commonly used term in the title or the abstract	1
		(*b*) Provide in the abstract an informative and balanced summary of what was done and what was found	2
**Introduction**			
Background/rationale	2	Explain the scientific background and rationale for the investigation being reported	3
Objectives	3	State specific objectives, including any pre-specified hypotheses	3
**Methods**			
Study design	4	Present key elements of study design early in the paper	3-5
Setting	5	Describe the setting, locations, and relevant dates, including periods of recruitment, exposure, follow-up, and data collection	3-4
Participants	6	(*a*) *Cohort study*—Give the eligibility criteria, and the sources and methods of selection of participants. Describe methods of follow-up<br>*Case-control study*—Give the eligibility criteria, and the sources and methods of case ascertainment and control selection. Give the rationale for the choice of cases and controls<br>*Cross-sectional study*—Give the eligibility criteria, and the sources and methods of selection of participants	3-4
		(*b*) *Cohort study*—For matched studies, give matching criteria and number of exposed and unexposed<br>*Case-control study*—For matched studies, give matching criteria and the number of controls per case	
Variables	7	Clearly define all outcomes, exposures, predictors, potential confounders, and effect modifiers. Give diagnostic criteria, if applicable	3-5
Data sources/ measurement	8*	For each variable of interest, give sources of data and details of methods of assessment (measurement). Describe comparability of assessment methods if there is more than one group	3-5
Bias	9	Describe any efforts to address potential sources of bias	5
Study size	10	Explain how the study size was arrived at	3
Quantitative variables	11	Explain how quantitative variables were handled in the analyses. If applicable, describe which groupings were chosen and why	3-5
Statistical methods	12	(*a*) Describe all statistical methods, including those used to control for confounding	5
		(*b*) Describe any methods used to examine subgroups and interactions	5
		(*c*) Explain how missing data were addressed	5
		(*d*) *Cohort study*—If applicable, explain how loss to follow-up was addressed<br>*Case-control study*—If applicable, explain how matching of cases and controls was addressed	5

For peer review only

		Cross-sectional study—If applicable, describe analytical methods taking account of sampling strategy	
		(*e*) Describe any sensitivity analyses	5
**Results**			
Participants	13*	(a) Report numbers of individuals at each stage of study—eg numbers potentially eligible, examined for eligibility, confirmed eligible, included in the study, completing follow-up, and analysed	6 and Fig 1
		(b) Give reasons for non-participation at each stage	Fig 1
		(c) Consider use of a flow diagram	Fig 1
Descriptive data	14*	(a) Give characteristics of study participants (eg demographic, clinical, social) and information on exposures and potential confounders	6, Table 1
		(b) Indicate number of participants with missing data for each variable of interest	Complete data only
		(c) *Cohort study*—Summarise follow-up time (eg, average and total amount)	
Outcome data	15*	*Cohort study*—Report numbers of outcome events or summary measures over time	
		Case-control study—Report numbers in each exposure category, or summary measures of exposure	
		Cross-sectional study—Report numbers of outcome events or summary measures	7, Table 1
Main results	16	(*a*) Give unadjusted estimates and, if applicable, confounder-adjusted estimates and their precision (eg, 95% confidence interval). Make clear which confounders were adjusted for and why they were included	8, Table 3
		(*b*) Report category boundaries when continuous variables were categorized	6, Table 1
		(*c*) If relevant, consider translating estimates of relative risk into absolute risk for a meaningful time period	n/a
Other analyses	17	Report other analyses done—eg analyses of subgroups and interactions, and sensitivity analyses	7,8
**Discussion**			
Key results	18	Summarise key results with reference to study objectives	6-11
Limitations	19	Discuss limitations of the study, taking into account sources of potential bias or imprecision. Discuss both direction and magnitude of any potential bias	13
Interpretation	20	Give a cautious overall interpretation of results considering objectives, limitations, multiplicity of analyses, results from similar studies, and other relevant evidence	12-14
Generalisability	21	Discuss the generalisability (external validity) of the study results	13
**Other information**			
Funding	22	Give the source of funding and the role of the funders for the present study and, if applicable, for the original study on which the present article is based	15

*Give information separately for cases and controls in case-control studies and, if applicable, for exposed and unexposed groups in cohort and cross-sectional studies.
Note: An Explanation and Elaboration article discusses each checklist item and gives methodological background and published examples of transparent reporting. The STROBE checklist is best used in conjunction with this article (freely available on the Web sites of PLoS Medicine at http://www.plosmedicine.org/, Annals of Internal Medicine at http://www.annals.org/, and Epidemiology at http://www.epidem.com/). Information on the STROBE Initiative is available at www.strobe-statement.org.

For peer review only
